# Unlocking the Potential of Marine Sidestreams in the Blue Economy: Lessons Learned from the EcoeFISHent Project on Fish Collagen

**DOI:** 10.1007/s10126-025-10438-9

**Published:** 2025-03-13

**Authors:** Lorenzo Dondero, Giulia De Negri Atanasio, Francesca Tardanico, Erica Lertora, Raffaella Boggia, Vittorio Capra, Agnese Cometto, Mattia Costamagna, Fi.L.S.E, Mirvana Feletti, Fulvio Garibaldi, Federica Grasso, Marte Jenssen, Luca Lanteri, Kjersti Lian, Marco Monti, Massimo Perucca, Cecilia Pinto, Ilaria Poncini, Federica Robino, Junio Valerio Rombi, Syed Saad Ahsan, Nikta Shirmohammadi, Micaela Tiso, Federica Turrini, Marta Zaccone, Matteo Zanotti-Russo, Ilaria Demori, Pier Francesco Ferrari, Elena Grasselli

**Affiliations:** 1 Department of Earth, Environment and Life Science, University of Genoa, Corso Europa 26, Genoa, Italy; 2 Angel Consulting, Via San Senatore 14, 20122 Milan, Italy; 3 Department of Pharmacy, University of Genoa, Viale Cembrano 4, 16148 Genoa, Italy; 4 National Biodiversity Future Center (NBFC), 90133 Palermo, Italy; 5 MICAMO Lab - Microbiologia Ambientale E Molecolare, Via XX Settembre 33/10, 16121 Genoa, Italy; 6 Ticass S.C.R.L.- Tecnologie Innovative Per Il Controllo Ambientale E Lo Sviluppo Sostenibile, Via Domenico Fiasella, 3/16, 16121 Genoa, Italy; 7 Project HUB-360, Corso Laghi 22, 10051 Avigliana, TO Italy; 8 Filse S.p.A., Piazza De Ferrari 1, 16121 Genoa, Italy; 9 Regione Liguria - Direzione Generale Turismo, Agricoltura E Aree Interne Settore Politiche Agricole E Della Pesca , Viale Brigate Partigiane, 2, 16100 Genoa, Italy; 10 Department of Marine Biotechnology, Nofima AS, Muninbakken 9-13, 9291 Tromsø, Norway; 11 Proplast, Via Roberto Di Ferro 86, 15122 Alessandria, AL Italy; 12 National Center for the Development of New Technologies in Agriculture (Agritech), 80121 Naples, Italy; 13 Department of Civil, Chemical and Environmental Engineering, University of Genoa, Via Opera Pia, 15, 16145 Genoa, Italy; 14 IRCCS Ospedale Policlinico San Martino, Largo Rosanna Benzi, 10, 16132 Genoa, Italy

**Keywords:** Blue Economy, Fish Collagen, Collagen in Cosmetics and Nutraceuticals, Collagen Biosynthesis, Collagen Extraction Methods, Packaging

## Abstract

This review provides a general overview of collagen structure, biosynthesis, and biological properties, with a particular focus on marine collagen sources, especially fisheries discards and by-catches. Additionally, well-documented applications of collagen are presented, with special emphasis not only on its final use but also on the processes enabling sustainable and safe recovery from materials that would otherwise go to waste. Particular attention is given to the extraction process, highlighting key aspects essential for the industrialization of fish sidestreams, such as hygiene standards, adherence to good manufacturing practices, and ensuring minimal environmental impact. In this context, the EcoeFISHent projects have provided valuable insights, aiming to create replicable, systemic, and sustainable territorial clusters based on a multi-circular economy and industrial symbiosis. The main goal of this project is to increase the monetary income of certain categories, such as fishery and aquaculture activities, through the valorization of underutilized biomass.

## Introduction

Sea sidestreams, originating from aquaculture and fisheries by-catches, constitute a source of several bioactive compounds, including polyphenols, polyunsaturated fatty acids, proteins, peptides, and polysaccharides. According to the UN agenda 2030 SDG Zero Hunger (Sustainable Development Goal), and given the decreasing terrestrial resources and the increase and aging of the global population, there is an urgent need to identify alternative sources of food and nutraceuticals. This is coupled with the necessity to increase certain groups’ monetary income, which cannot benefit from substantial profits at the beginning of the production chain.

Marine-derived collagen and its derivatives, such as gelatin and hydrolyzed collagen (HC), are well characterized and consist of versatile molecules offering several advantages, such as application in multiple fields without any religious and hygienic constraints. The use of sidestreams for human-oriented applications raises some concerns about managing the valorization chain in accordance with good manufacturing practices; these are aimed to guarantee the control of several parameters, such as preventing the introduction of risk to humans. Moreover, within the sustainability framework, it is mandatory that all the actions within the entire chain, aimed at the valorization of sidestreams, are checked from a life cycle assessment (LCA) point of view to verify the sustainability of these products from the cradle to the grave.

This review, after a general overview of collagen structure, biosynthesis, and biological properties, is focused on marine collagen sources, particularly fisheries discards and by-catches. Further, applications of collagen are considered, with particular attention to the processes underlying a sustainable and safe recovery from fisheries sources that would otherwise be wasted.

In this context, the example of the EcoeFISHent project is described (Horizon 2020, No 101036428, https://ecoefishent.eu), which is centered in Italy, specifically in the Liguria region, and focuses on collagen from marine sidestreams of the Mediterranean Sea, serving as an example of sustainability linked to marine activities. The project aims to demonstrate a replicable, systemic, and sustainable territorial cluster based on a multi-circular economy and industrial symbiosis.

### Collagen: The Basic Structural Model and Types

The word “collagen” derives from the ancient Greek “kolla” (κόλλα) and “genes” (γενήϛ), literally meaning “glue generator.” Collagen is a key protein in the human body, responsible for the structure and integrity of connective tissues such as the skin, bones, cartilage, and others. In this regard, collagen is the main component of the extracellular matrix, primarily serving as a site for cell adhesion, thus ensuring the integrity of tissues and organs. Moreover, it can promote cellular regeneration and aid wound healing. Finally, it also contributes to skin health by keeping it elastic and hydrated, supporting joint flexibility, and giving structure to the blood vessels and internal organs. With aging, collagen production may decrease, affecting the health of the skin, joints, and other connective tissues (Lim et al. 2019a).

**Fig. 1:**
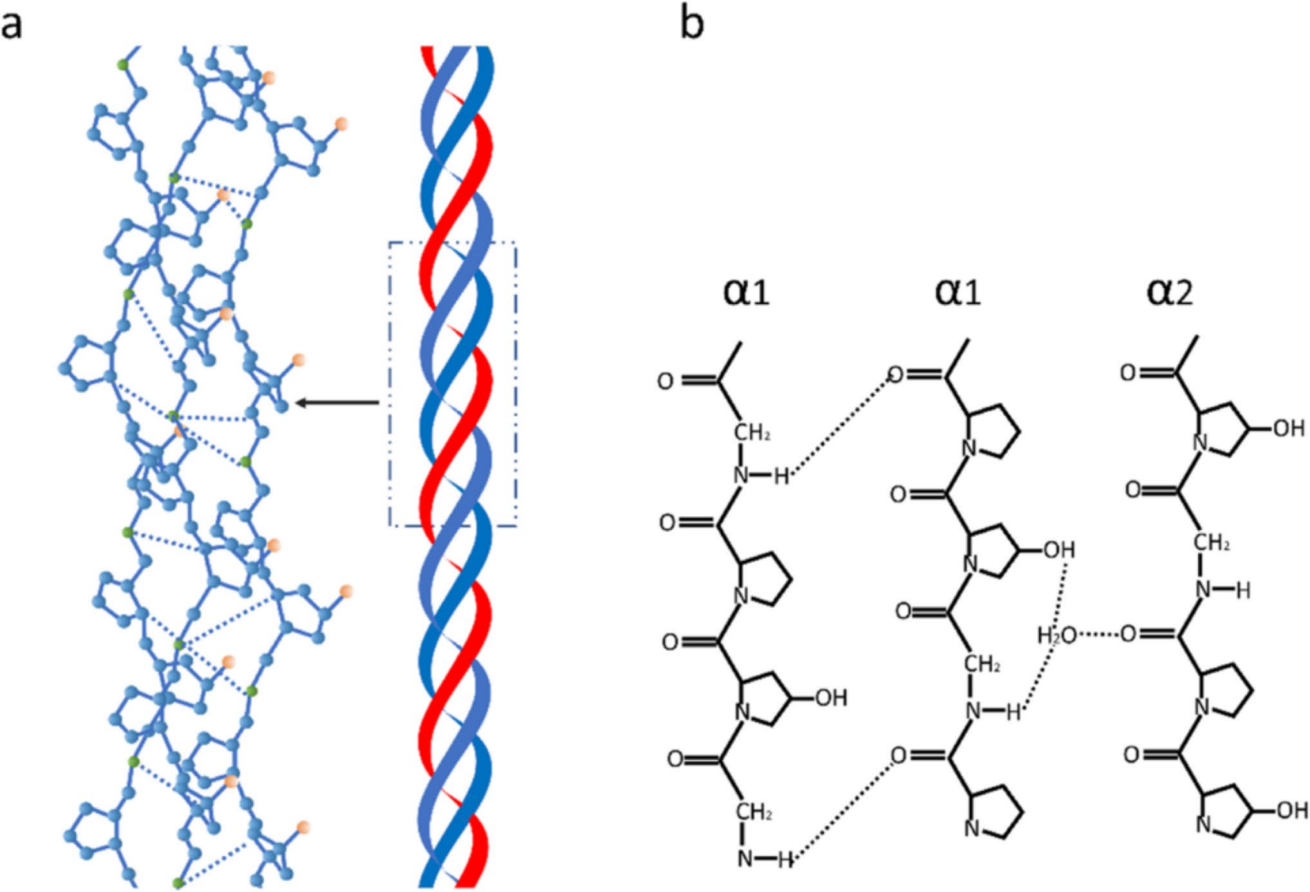
**A** The triple helix of type I collagen. One procollagen I α2 subunit pairs with two procollagen I α1 subunits to generate type I procollagen. The N and C terminal propeptides are removed proteolytically to produce mature type I collagen. **b** Hydrogen bonds of the collagen model. Every α-chain relates to each other through hydrogen and covalent bonds, which give collagen a high level of strength. All members of the collagen family could form these supramolecular structures, although their size, function, and tissue distribution vary considerably (Van Der Rest and Garrone 1991a). Indeed, the diversity of the collagen family is further modified thanks to the presence of different α-chains, different molecular isoforms, and supramolecular structures for a single collagen type, due to the presence of different promoters and alternative splicing occurring during collagen synthesis (Zhang et al. 2020)

All collagen molecules share a distinctive structure comprising a triple helix formed by three polypeptide chains (Jeevithan et al. 2013) (Fig. 1a).

This triple helix can be made up of three identical polypeptide chains, known as homotrimers (as in type II collagen), three different chains, or two identical chains and a third distinct one heterotrimers (type I collagen).

Moreover, the triple helix can range from most of their structure (96% for collagen type I) to less than 10% for collagen type XII (Fraser and Tenner 2008). These chains are looped around each other to form a tight and stable structure (León-López et al. 2019a) featuring a repeating triplet of proline (Pro) (22%), glycine (Gly) (33%), and hydroxyproline (Hyp) (14%) (Eastoe 1957), which also provides a distinctive marker of this protein (Fig. 1b) (Eastoe 1957).

Two hydrogen interchain bonds (Baselt et al. 1993; Gale et al. 1995; Peñuela et al. 2018) *per* triplet are found: one between the amine group of a glycyl residue and the carboxyl group of the residue in the second position of the triplet in the adjacent chain and one via the water molecule participating in the formation of additional hydrogen bonds with the help of the hydroxyl group of hydroxyproline in the third position (Baselt et al. 1993; Gale et al. 1995; Peñuela et al. 2018).

**Fig. 2:**
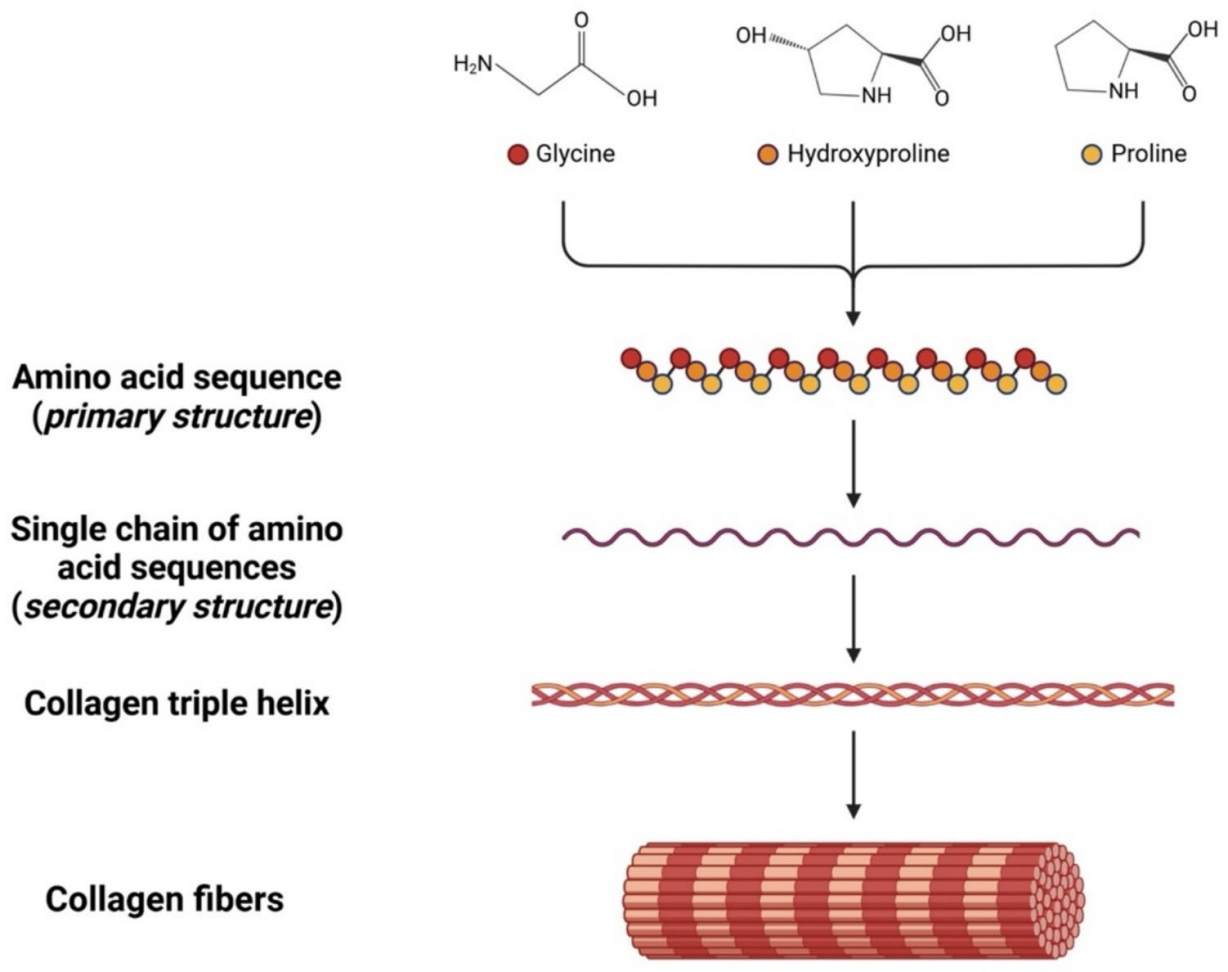
Schematic representation of collagen structural organization levels. Collagen protein is organized into four structural levels, including amino acid sequence; the α-helix single chain of amino acid sequences; collagen triple helix, composed of three α-helix; collagen fibers, formed by several triple helices joined together (Gelse 2003a). Every type of collagen can be distinguished by the complexity and diversity of the structure, such as the splice variants, the presence of additional, non-helical domains, and their assembly. Each collagen type has a specific alpha chain with its particular domain structure that contributes to distinguishing each type from the others (Coppola et al. 2020). The figure was created using BioRender (Onursal et al. 2021) (https://pdb101.rcsb.org/motm/4)

As shown in Fig. 2, collagen protein is organized into four structural levels; the entire collagen structure has a molecular weight of approximately 350 kDa (Sorushanova et al. 2019). Specifically, each α-helix chain comprises roughly 1014 amino acids with a molecular weight of around 100 kDa (León-López et al. 2019a), a length of around 300 nm, and a diameter of about 1.4 nm. In addition to the triple helical region, there are two non-helical regions at either end of the helix structure called “non-collagenous” (NC) domains, and they are numbered from the C-terminus (NC1, NC2, etc.) (Schrieber and Gareis 2007a).

**Fig. 3:**
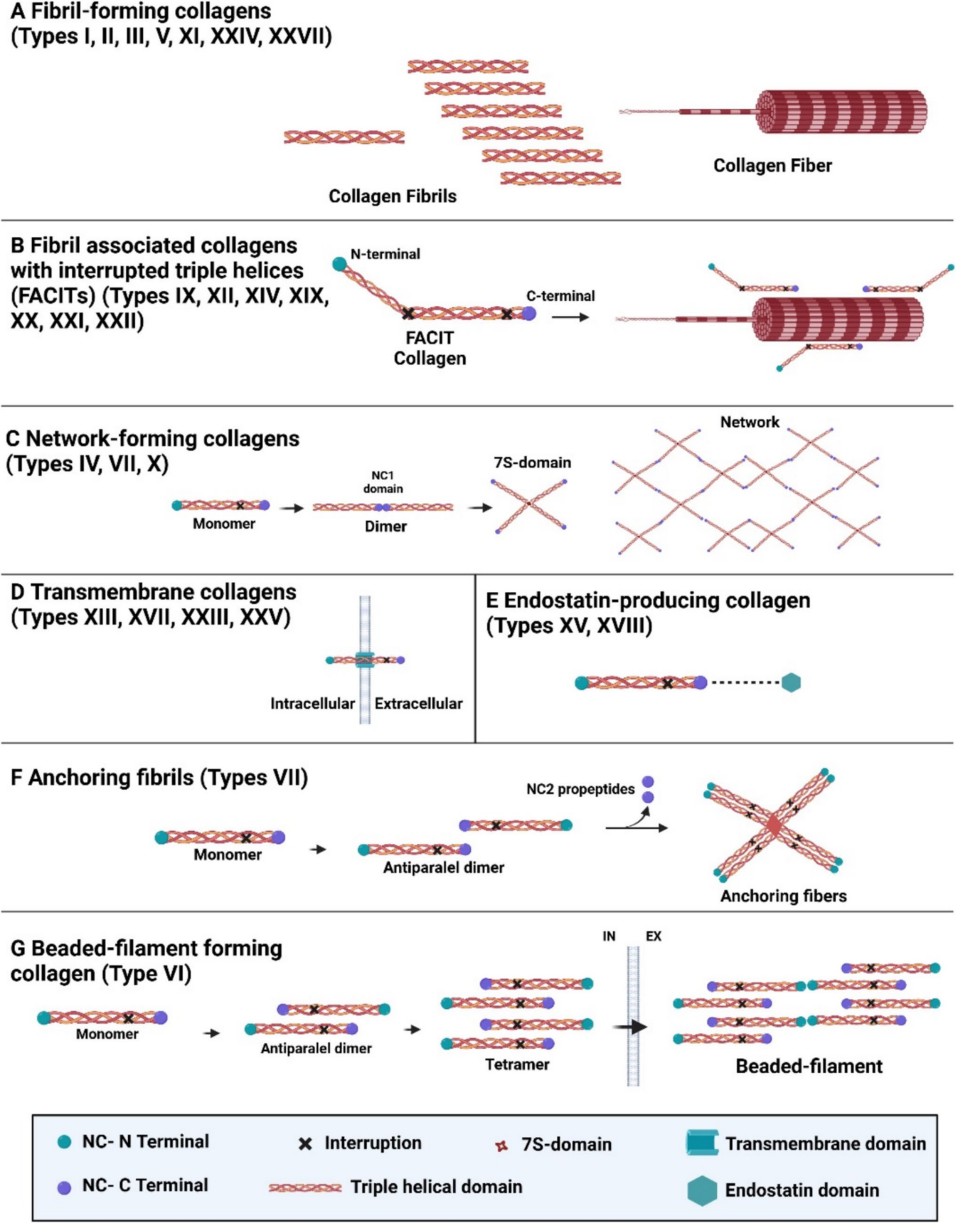
Classification of collagens based on supramolecular assembly. Collagen molecules are grouped into several families depending on their structure, chain bonding, and position in the human body, but most of the collagen types belong to the fibril forming (80–90% of total collagen) of the fibrillar family. Inside this classification, it is possible to find the fibril forming (A) (I, II, III, V, XI, XXIV, XXVII), fibril-associated collagens with interrupted triple helix (B) (FACIT, IX, XII, XIV, XVI, XIX, XX, XXI, XXII), network-forming collagens (C) (IV, VIII, X), transmembrane collagens (D) (XIII, XVII, XXIII, XXV), endostatin-producing collagen (E) (XV, XVIII), anchoring fibrils (F) (VII), and beaded filament-forming collagen (G) (VI) (Gelse 2003b; Fratzl 2008; (Holmes et al. 2018; Kadler et al. 1996). The image was created using BioRender (https://www.biorender.com/)

Currently, it is known that vertebrates have 40 collagen genes, which make up the entire collagen family consisting of 29 different types of molecules; the majority are more interrelated and confined to specific tissue locations. This variety signifies a range of biological roles manifested through numerous physical formations (Fig. 3) (Sorushanova et al. 2019).

The first three types of collagens constitute the extracellular matrix of many organs and tissues. Type I collagen is the most abundant in the human body, bones, tendons, and dermis. In the latter, collagen type I constitutes about 80–90% of the total collagen and maintains the mechanical strength of the skin along with its elasticity (Fratzl 2008). One peculiarity of the collagen molecule is represented by a dextrorotatory helix consisting of three levorotatory alpha helices, thus resulting in tropocollagen configuration (Kadler et al. 2007). Type II collagen is predominant in cartilage, forming a three-dimensional network (fibrils) that provides structural support and contributes to compression resistance. This is crucial for joint function, ensuring resistance to pressure during movement (Eyre and Wu 2005). Type III collagen is found in elasticized tissues such as blood vessels, lungs, and skin. Its reticular structure allows tissues to stretch and contract, providing the flexibility necessary to adapt to physiological demands. This characteristic is crucial for the functioning of the cardiovascular system and the maintenance of the skin’s structural integrity (Kadler et al. 1996).

Besides fibroblasts, which are the main producer of collagen in the connective tissues (Matsuda et al. 2006), other cells (i.e., hepatocytes (Clement et al. 1986), adipocytes (Friedman 1990), osteoblasts (Friedman 1990; Rossert and de Crombrugghe 2002)) are involved in the production of certain types of collagens. Fibroblast activation and proliferation can be induced by physical and chemical stimuli. Generally, chemical stimuli are based on a “key-lock” mechanism: some receptors located on the fibroblast membrane are bound to small ligands to induce their activation. Otherwise, physical stimuli are directly related to the interactions between collagen and fibroblasts (Narayanan et al. 1989).

### Collagen Biosynthesis

**Fig. 4:**
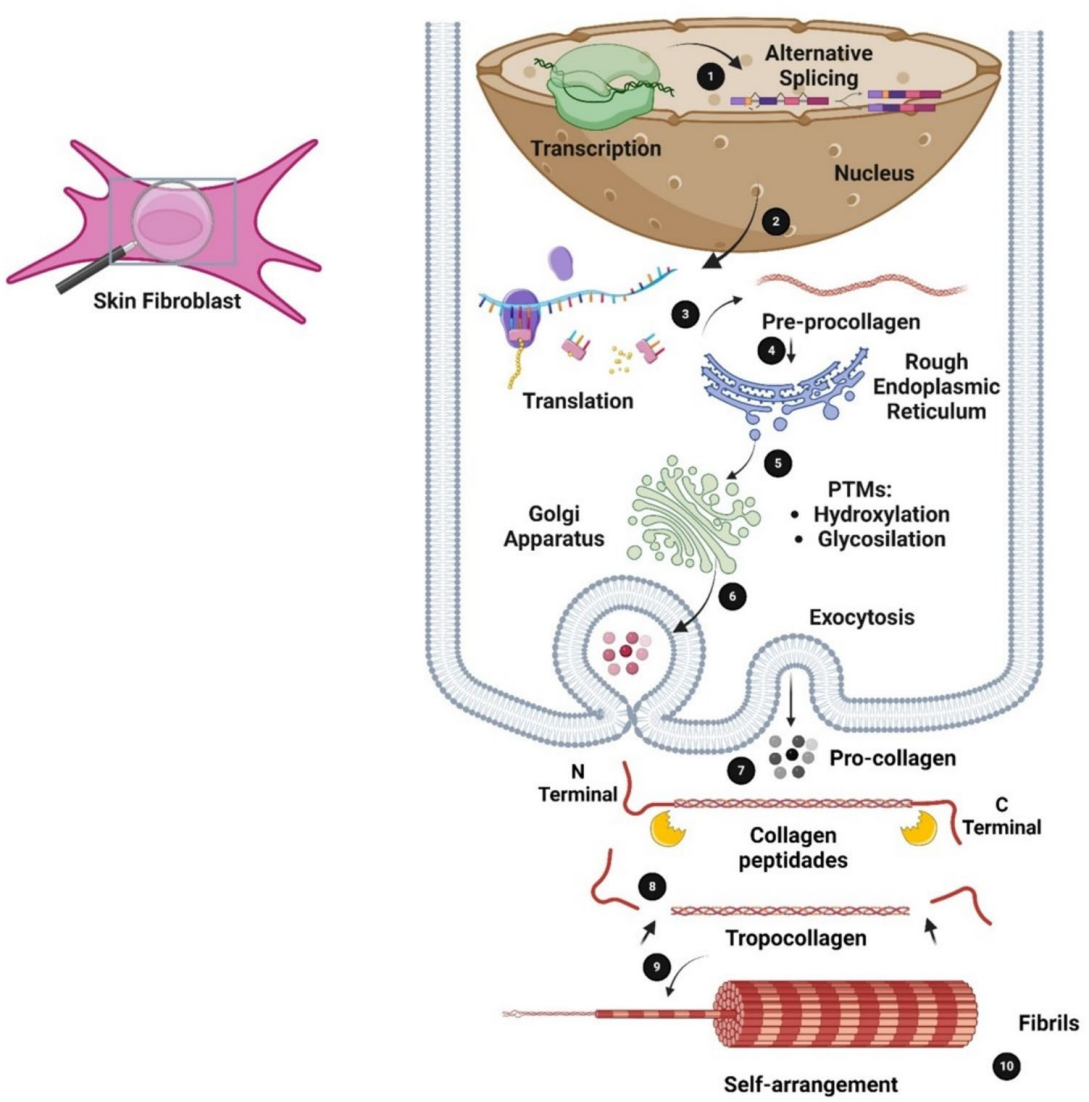
Intracellular collagen biosynthesis and extracellular maturation of type I collagen. (1) Transcription and alternative splicing of the mRNA. (2) The mRNA leaves the nucleus through the nuclear pores and reaches the cytosol. Translation (3) and translocation of the nascent polypeptide chain to the rough endoplasmic reticulum (RER) (4). Activation of the two major post-translational modifications (PTMs) of collagen in the E: Hydroxylation and glycosylation (5); and folding of the C- and N-terminal propeptides (C and NT) to form collagen triple helix. This is followed by transport via the trans-Golgi network (6) and secreted into the extracellular space (7). Extracellular processing and maturation of the triple helix of collagen (8), where propeptide cleavage involves at least three proteases, triggers the auto-assembly of collagen fibrils (9). Finally, fibrils are stabilized by cross-linking (10) (Onursal et al. 2021). The figure was created using BioRender (https://www.biorender.com/)

Collagen biosynthesis is a highly complex process that starts with gene transcription followed by translation and translocation of the nascent polypeptide chain to the rough endoplasmic reticulum (RER), co-translational modification and folding, trafficking across the Golgi network, secretion, and finally, extracellular processing and maturation (Fig. 4). This pathway requires the coordination of numerous temporally and spatially coordinated biochemical events.

Starting from fibroblast cells, depending on the collagen type and isoform, the initial step of the intracellular biosynthesis of collagen involves the transcription of mRNA molecules encoded by various three α-chain genes (Bailey and Paul 1998).

In humans, the α1 chain of type I collagen is encoded by a gene located on the long arm of chromosome 17 (17q21.31q22). The α2 chain is encoded by a gene found on the long arm of chromosome 7 (7q21.3-q22) (de Wet et al. 1987; D’Alessio et al. 1988). The triple helical domain of the α1 chain is encoded by 41 exons and the α2 chain by 42 exons (Rossert and de Crombrugghe 2002). The nascent collagen α-chain enters the lumen of the RER with the N-terminus first as pre-procollagen, which is converted into procollagen molecule by the removal of the signal peptide (Fig. 4) (Sorushanova et al. 2019).

After being transcribed, the pre-mRNA undergoes exon splicing, capping, and addition of a poly(A)tail, giving rise to a mature mRNA that can be translated into polysome. The synthesized protein then undergoes extensive post-translational modifications (PTMs) before assembling into a triple helix and being secreted into the extracellular space (Rossert and de Crombrugghe 2002).

It is important to note that biosynthesis starts at the N-terminus, while triple-helix formation starts at the C-terminus. For this reason, the pro-α-chain needs to remain untangled until the α-chain translation is completed, and three chains align precisely at the C-terminus before triple-helix formation begins. Numerous chaperone proteins play a crucial role in avoiding α-chain entanglement: these include prolyl 4-hydroxylase (P4-H), a counterpart of the RER’s heat shock protein (hsp) 70 (BiP/Grp78), as well as hsp47 which binds to procollagen until it reaches the Golgi apparatus, where it subsequently detaches probably due to a pH change (Sorushanova et al. 2019). At body temperature, more than 20 hsp47 molecules are needed *per* triple helix to stabilize the protein (Ricard-Blum 2011).

Additionally, two major PTMs of collagen are hydroxylated and glycosylated in the RER. These contribute to an increase in the thermal and mechanical stability of the triple helix and its assembled form. Pro undergoes hydroxylation to become Hyp through the enzymatic action of P4-H, located in the RER of fibrogenic cells. This amino acid is essential to form interhelical hydrogen bonds within the triple helix, which provides thermal stability, essential at body temperature. For this reason, in organisms with warm-blooded metabolisms, a minimum of 100 Pro residues *per* pro-α-chain must be converted to Hyp to guarantee the necessary thermal stability (Privalov et al. 1979).

Lysine (Lys) is also hydroxylated, resulting in hydroxylysine (Hyl), an amino acid that, in conjunction with the attachment of sugar components, modulates fibrillogenesis and fibril stability by covalent cross-linking.

Following the PTMs, the arrangement of the three pro-α-chains, termed “registration,” is guided by the C-propeptides. The C-propeptides contain cysteine residues responsible for the sole covalent bonds within the procollagen trimer—disulfide bonds—which are lost upon removal of the propeptides during secretion, while intracellularly, they ensure a secure alignment between α-chains. On average, it takes about 14 min for a procollagen type I triple helix to fold.

The procollagen molecule is transported to the Golgi apparatus, where glycosylation is finalized before it is enveloped in vesicles and released into the extracellular space where the procollagen molecules undergo cleavage by N- and C-procollagen-specific enzymes, such as metalloproteinases. Then, the molecules can fold into a triple-helix structure, leading to a mature collagen molecule (tropocollagen) (Kessler et al. 1996).

The newly formed tropocollagen molecules spontaneously aggregate to form collagen fibrils. This process in vivo is dependent on the involvement of cells that interact with nascent and mature fibrils through cell surface receptors, such as integrins and the glycoprotein fibronectin, often referred to as “fibril organizers.” Fibronectin forms a fibril network, which is subsequently engaged by integrins, acting as a template for further collagen fibril assembly.

Collagen fibrils, with their highly organized structure, act as the substrate for lysyl oxidase. This extracellular enzyme facilitates the oxidative deamination of specific Lys and Hyl residues within collagen, producing reactive aldehydes (such as allysine and hydroxyallysine). These undergo condensation reactions with Lys or Hyl residues, leading to covalent cross-links between chains. The cross-linking is crucial for attaining the tensile strength necessary for the proper functionality of connective tissue, since it ensures that the tissue can withstand mechanical stress and maintain its integrity in various physiological contexts (Sorushanova et al. 2019). Finally, the fibrils can arrange themselves in wavy or parallel bundles to form fibers, and those can, in turn, form fiber bundles. The paring occurs between a stretch of 234 amino acids of either helix, a region that ensures maximal electrostatic interaction and hydrophobic interactions.

The hierarchical assembly of collagen molecules imparts structural stability, mechanical integrity, and enzymatic resilience to collagen-based tissues. This structural integrity is further reinforced by both weak interactions and robust intermolecular cross-links. For instance, type I collagen achieves stabilization through the presence of four cross-links, two located in the helical region and an additional one in each telopeptide (Smith-Mungo and Kagan 1998).

Collagen fibrils are generally made of different collagen types, for example, type I and III collagens in the skin and type II and III collagens in the cartilage. Collagens are also able to be bundled into a large fascicle with several hundreds or thousands of fibrils and other additional molecules bound to their surfaces, such as glycoproteins, proteoglycans, plasma membrane receptors including integrins, discoidin domain-containing receptors (DDRs), and mannose receptors (Holmes et al. 2018). The fibril diameters, the fibril volume fractions, and the spatial arrangement of fibrils depend on the tissue and stage of development.

As far as it is known, the biosynthetic pathways for all the different collagen types are quite overlapping. However, the differences among collagen types originate from their primary structure, which, in turn, depends on the gene sequences encoding for the different types. PTM steps can also contribute to further differentiating collagen types (Last and Reiser 1984).

### Collagen Derivatives: Gelatin and Hydrolyzed Collagen

Gelatin and hydrolyzed collagen can be produced from collagen. These have different structures and molecular weights, leading to physicochemical properties distinguishable from the native collagen. The triple-helix structure of native collagen (tropocollagen) is preserved, while gelatin consists of denatured native collagen. The molecular weight of gelatin ranges from 94 kDa (average molecular weight of type A gelatin) to 171 kDa (average molecular weight of type B gelatin) (Haug and Draget 2009). Moreover, collagen/gelatin hydrolysates HC derive from an enzymatic process that hydrolyzes the peptide bonds of the native molecule to obtain small peptides with low molecular weight (0.3–8 kDa), produced from native collagen. The terms “hydrolyzed gelatin,” “collagen hydrolysate,” “hydrolyzed collagen,” or sometimes “collagen peptides” used in publications describe the same product. Gelatin is obtained by partial thermal hydrolysis of collagen, which partially separates the chains by destroying the cross-links (Daneault et al. 2015). Upon hydrolysis, the breaking of peptide bonds implies the fragmentation of collagen molecules leading to the disruption of hydrogen bonds within collagen helices. The hydrolyzed molecules cannot maintain the native structure and assume random coil conformation. The molecular weight of these collagen peptides obtained through hydrolysis is considerably lower, typically ranging from 3 to 6 kDa (León-López et al. 2019b), compared to the precursor native collagen, which has a molecular weight of 285–300 kDa and 1.4 nm in diameter (Gelse 2003b; Sorushanova et al. 2019; Schrieber and Gareis 2007b).

Gelatin (Ahmad et al. 2024) is a heterogeneous mixture of water-soluble collagenic proteins divisible into two types: type A and type B, linked to the extraction process. Type A gelatin (positively charged) refers to acid extraction, and type B gelatin (negatively charged) refers to alkaline extraction. Upon the different extraction processes, the generated isoelectric point (pI) can enhance the binding between gelatin and the charged therapeutic agents. The pI for type A and B gelatins is usually about pH 9 and 5, respectively (Naomi et al. 2021). Collagen, being an amphoteric macromolecule, exhibits a pI value typically ranging from 7 to 8. During the hydrolysis process, the pI value of collagen undergoes a shift towards lower values, typically ranging from 3.68 to 5.70. The extent of this shift depends on various factors, such as the specific amino acid sequences present in collagen and the distribution of amino acid residues. The type and duration of hydrolysis also influence this change in the pI value (Chen et al. 2016). Enzymatic hydrolysis affects the size of the peptides and their physicochemical and biological properties (Zhang et al. 2017).

Regarding solubility, collagen is insoluble in water, but it is possible to solubilize it in diluted acids or bases; on the other hand, gelatin is soluble in water and can form a gelatinous solution when cooled (Gómez-Guillén et al. 2011). The solubility of HC is variable since it depends on several factors influencing the composition, such as animal source (i.e., terrestrial, marine), tissue (i.e., skin, tendons), and the extraction process (León-López et al. 2019b).

Viscosity is a significant physicochemical property of collagen. In its native form, collagen exhibits higher viscosity, which can be attributed to the stronger electrostatic repulsion between the molecular chains, even at low concentrations. This repulsion contributes to the resistance encountered by the flowing solvent. On the other hand, HC demonstrates significantly reduced viscosity, irrespective of its concentration. This decrease in viscosity is primarily due to the low molecular weight of the small chain segments obtained through hydrolysis. The smaller chains have less capacity to interact and entangle with each other, resulting in reduced resistance to flow and, subsequently, lower viscosity (Sun Pan et al. 2018). The primary property of gelatin is its ability to form temperature-reversible gels via the formation of a three-dimensional network by chemical cross-linking (Said and Sarbon 2022). The viscosity of a gelatin solution depends on concentration, temperature, and bloom value, which describes the gel strength and serves as an index of the strength and stiffness of the gelatin. Both bloom value and viscosity of gelatin could vary depending on the animal source, environment, and extraction method used. A comparison of gelatins derived from terrestrial and marine organisms was documented by Leuenberger (1991), demonstrating that fish gelatins have a lower gelation temperature but relatively high solution viscosities when compared to those derived from terrestrial organisms. Fish gelatin generally has a lower bloom value compared to mammalian gelatin (Said and Sarbon 2022). The bloom values for marine gelatins can vary more widely because of the varying levels of Pro and Hyp in collagens from different species and the temperatures of their habitats (Ninan et al. 2014).

Both gelatin and hydrolyzed collagen peptides find their application in numerous fields. Gelatin can be obtained at a very low cost and has a high range of biodegradability (Mohiti-Asli and Loboa 2016). The versatility of gelatin depends on its thickening and gelling properties, making it useful in various industries beyond the culinary field (Deshmukh et al. 2017). The drying temperature of gelatin influences its properties, and it possesses a perfect film-forming activity. This is why gelatin is widely used as a drug delivery agent. Indeed, in the pharmaceutical industry, gelatin is used to produce capsules and coatings for medications as it is a safe and easily digestible substance. Moreover, gelatin acts as a barrier to gas and hydrophilicity, even if it has poor mechanical strength. Gelatin is also used as a thickening agent in cooking and food production. In cosmetics, gelatin is utilized in skincare and hair care products. It can be found in creams, facial masks, shampoos, conditioners, and other products to provide texture and consistency or act as a moisturizing agent.

The composition and degree of collagen hydrolysis can increase some functional properties of collagen, such as antioxidant capacity, antimicrobial activity, and higher bioavailability. All these properties are mainly related to the molecular weight value, which allows HC to produce more stable products reacting with free radicals (Wang et al. 2018). HC offers numerous advantages over native collagen, making it a preferred choice in various applications. For example, HC has a lower molecular weight, which allows for greater bioavailability and better absorption, enhancing its therapeutic potential. Its production is more cost-effective than native collagen due to a simplified hydrolysis process. This makes HC highly digestible and easily absorbed. Moreover, in the dermis, HC supplies amino acids for collagen and elastin formation and stimulates fibroblasts to produce new collagen, elastin, and hyaluronic acid, making it ideal for cosmetic applications (Sibilla et al. 2015; Avila Rodríguez et al. 2018).

## Collagen Sources

**Fig. 5:**
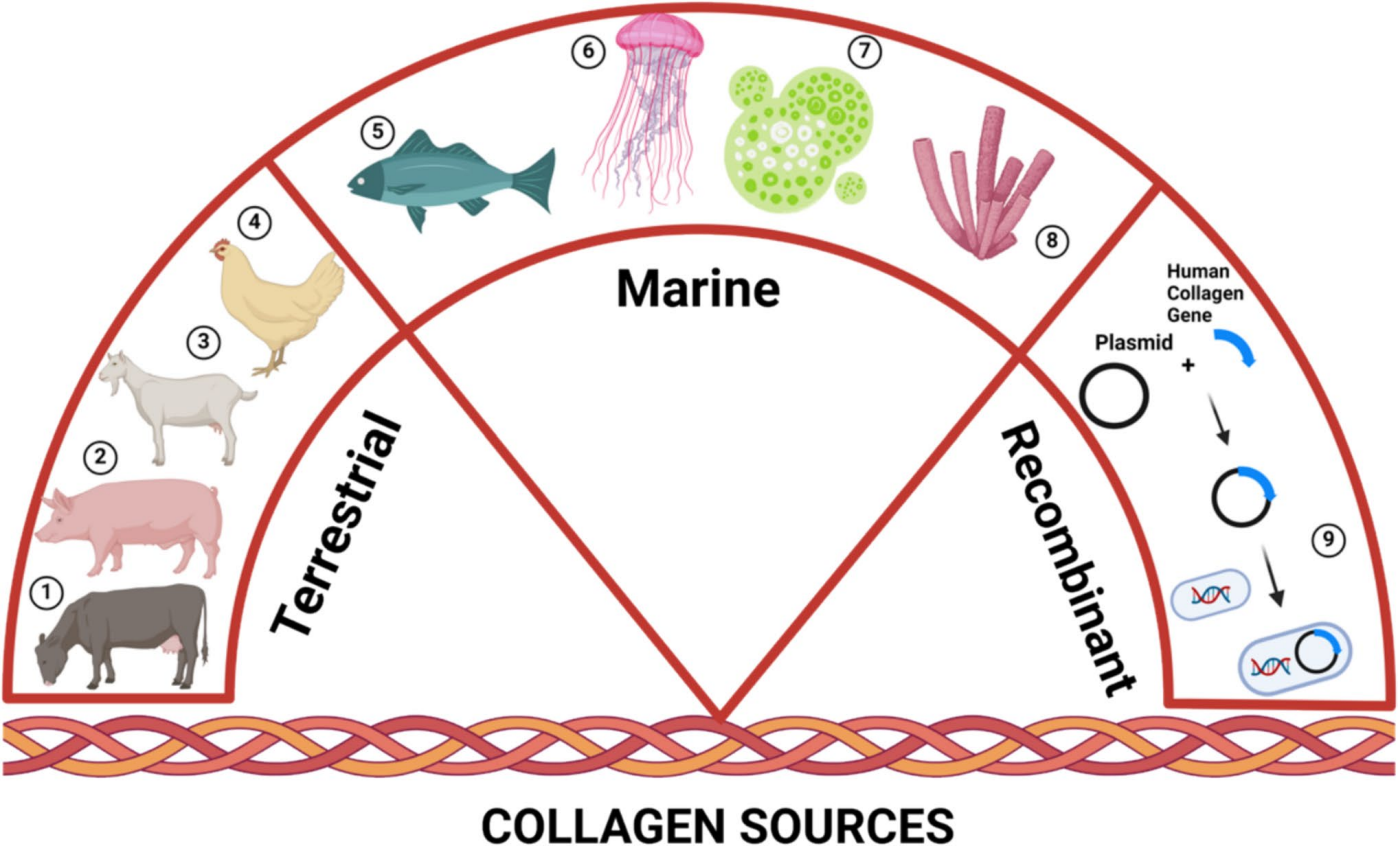
The bones and skin of terrestrial animals such as cow (1), pig (2), goat (3), and chicken (4) are the main sources of collagen. Fishes (5) are a valuable collagen source, in recent decades, invertebrates like cuttlefish, sea anemones, prawns, starfish, jellyfish (6), microalgae (7), sponges (8), sea urchin, octopus, squid, and mollusks are gaining attention as potential collagen sources (Xu et al. 2010). Another possible option is to employ recombinant DNA technology (9) as an alternative method to obtain non-animal-derived collagen; this technique could represent a scalable and repeatable alternative, although this source is still under development (Martínez-Puig et al. 2023). The image was created using BioRender (https://www.biorender.com/)

Collagen can be extracted from different sources, such as vertebrates and invertebrates. The most common terrestrial sources are cattle, pigs, poultry (Avila Rodríguez et al. 2018), and marine organisms (particularly fish scales (Fan et al. 2013) and fish skin; Karami et al. 2019; Akram and Zhang 2020; Yousefi et al. 2017; Silvipriya et al. 2015) (Fig. 5).

One of the most common sources used to isolate bovine-derived collagen type I is the Achilles tendon (Ahmed et al. 2020). The amount and solubility of collagen obtained vary with the age of the animal, with younger tissues yielding more collagen (Tanase et al. 2011). HC from porcine skin and bones is also a traditional source commonly utilized for industrial purposes. Its similarity to human collagen makes it safe, avoiding allergic responses. With a low molecular weight (1–10 kDa), it exhibits antioxidant, anti-aging, skin permeation properties, and ACE inhibitory potency (Choi et al. 2018; Ahmed et al. 2020).

Concerns over the increased risk of human infections from bovine spongiform encephalopathy (BSE), foot and mouth disease (FMD), and transmissible spongiform encephalopathy (TSE) from cows and pigs have prompted the exploration of alternative animal sources for collagen production. Moreover, ethical, religious, and social issues limit the utilization of certain animal sources for collagen extraction. For example, some populations are subject to rules that limit the consumption and/or usage of bovine or porcine derivatives. Consequently, the marine environment appears to be a promising new alternative collagen source. Marine collagen, derived from fish or other marine organisms, has gained significant attention in recent years. Type I fish skin collagen has shown great potential as a substitute for mammalian collagen sources (Silva et al. 2014). The collagen yield from fish sidestreams can exceed 50% in dry mass, making it an eco-friendly and cost-effective source. Marine collagens from both vertebrates and invertebrates present an increase in bioavailability compared to the traditional sources (bovine and porcine) thanks to a greater possibility of absorption, to the low molecular weight, and to the small particle size, which can circulate rapidly through the bloodstream (Jafari et al. 2020).

In addition to the extraction from animal tissues, including marine organisms (Sibilla et al. 2015), collagen can be generated from recombinant protein production systems using bacteria, yeasts, insects or plants, mammalian cells, or artificial fibrils (Rodríguez et al. 2017). In Fig. 5, all the collagen sources that are currently employed are described.

## Marine Collagen Sources from Discard and By-Catch of the Ligurian Sea

“Discards or discarded catch is that portion of the total organic material of animal origin in the catch, which is thrown away, or dumped at sea for whatever reason. It does not include plant materials and post-harvest waste such as offal. The discards may be dead, or alive” (www.fao.org). The discard management from natural sources (like fisheries) and human-made (such as marine litter) is becoming increasingly important with the implementation of EU Regulation No. 1380/2013. This regulation includes Article 15, known as the landing obligation, or the discard ban, which mandates the gradual elimination of species below the minimum catch reference size (MCRS) to tackle the problem of discards at sea. In Italy, the de minimis exemption under the EU Regulation 1380/2013 is in force, as it has been shown that the average discard of species subject to the landing obligation is below 5% of the annual total landing. Planning and future scenarios regarding discards are still evolving (see Regulation 2306/2018 of 18 October 2018). Numerous projects (PRISMAMED project, the Project National Center for the Development of New technologies in Agriculture (Agritech) Project code CN 00000022, the Project National Biodiversity Future Center (NBFC), Project code CN 00000033, and EcoeFISHent) have been initiated to enhance the utilization of fisheries resources by obtaining bioactive elements from discards.

The Ligurian Region, in the framework of the PRISMAMED (“Piano RIfiuti e Scarti in Mare di pesca, acquacoltura e di porto nel Mediterraneo 2018–21”; https://interreg-maritime.eu/web/prismamed) project, aimed to develop managing strategies of discard from fishing activities to give possible solutions for reusing and recycling discarded products in a circular economy perspective. One of the objectives was to characterize qualitatively and quantitatively the discard fraction (organic and inorganic) produced by the trawling activity of Santa Margherita Ligure (SML) (Liguria, Italy) fishing fleet. The fleet operates on two separate areas: the continental shelf (SHE, between 50 and 200 m depth) and the continental slope (SLO, 200–800 m depth). Many different factors can affect the discard composition depending on the biology and ecology of the species (habitat, depth, season, size, assemblage), the fishing technique (gear dimension, mesh size, vessel capacity, effort, fishers’ behavior), and the market demands (Rochet et al. 2014; Carpentieri 2019).

Within the PRISMAMED project, a total of 113 species were classified: 64 OS (bony fish), 17 CR (crabs and shrimps), 11 MO (cephalopods, gastropods, and nudibranchs), 6 CH (sharks, rays, and chimeras), 6 EC (starfish and sea cucumbers), 5 of CN (soft/hard corals and jellyfish), 3 PO (sponges), and a single species of TU (sea squirt). Fifty-seven percent of the species (*n* = 64) were classified as target or commercial, while 43% of the species (*n* = 49) had no commercial value or were not edible species. Seventy-one species were sampled in the shelf area, while 50 species were sampled on the continental slope, and 8 species were common to both fishing areas. Twelve species (11 OS and 1 CR) were detected among those with a MCRS. Undersized individuals constituted 75% of the overall discard in weight (256 kg) and were in large part composed of juvenile specimens. The OS represents about 84% of the overall discard in weight (288.3 kg; 2.4 kg/h) followed by CH (26.1 kg; 0.22 kg/h) and CR (11.1 kg; 0.09 kg/h).

Considering the two different fishing areas separately, it is evident that most of the discard is represented within the shelf area catches, except for CH and MO (Table 1), mostly represented by non-commercial species or, as for blackmouth catshark (*Galeus melastomus*), landed on market demand. The blackmouth catshark is documented to be a valuable source of both (Karayannakidis et al. 2024) collagen and chondroitin sulfate (Vázquez et al. 2018), representing a way to valorize by-catch and further confirming the possibility of using by-catch as a source of bioactive molecules. As extensively reviewed in other studies, the type of collagen and its amino acid sequence can vary depending on the source within the same species (such as the skin, bone, cartilage, and scales) and/or across different species. These variations result in different physicochemical properties, leading to diverse applications.

**Table 1:** Discard and catch rate observed over the shelf (SHE) and slope (SLO) area*

Taxa	SHE			SLO			Total		
	**Discard (kg)**	**Catch rate (kg/h)**	**%**	**Discard (kg)**	**Catch rate (kg/h)**	**%**	**Discard (kg)**	**Catch rate (kg/h)**	**%**
OS	273.86	4.52	91.82	14.42	0.24	33.37	288.27	2.39	84.42
CH	3.67	0.06	1.23	22.45	0.37	51.96	26.12	0.22	7.65
CR	8.41	0.14	2.82	2.70	0.05	6.26	11.11	0.09	3.25
EC	4.60	0.08	1.54	0.08	0.00	0.18	4.68	0.04	1.37
MO	1.09	0.02	0.37	3.41	0.06	7.90	4.50	0.04	1.32
TU	3.59	0.06	1.20	-	-	-	3.59	0.03	1.05
PO	2.37	0.04	0.79	0.03	0.001	0.08	2.40	0.02	0.70
CN	0.69	0.01	0.23	0.11	0.002	0.26	0.80	0.01	0.23

*The data presented in this table consist of unpublished data from the PRIMSAMED project

## Extraction of Collagen and Hydrolyzed Collagen Peptides

**Fig. 6:**
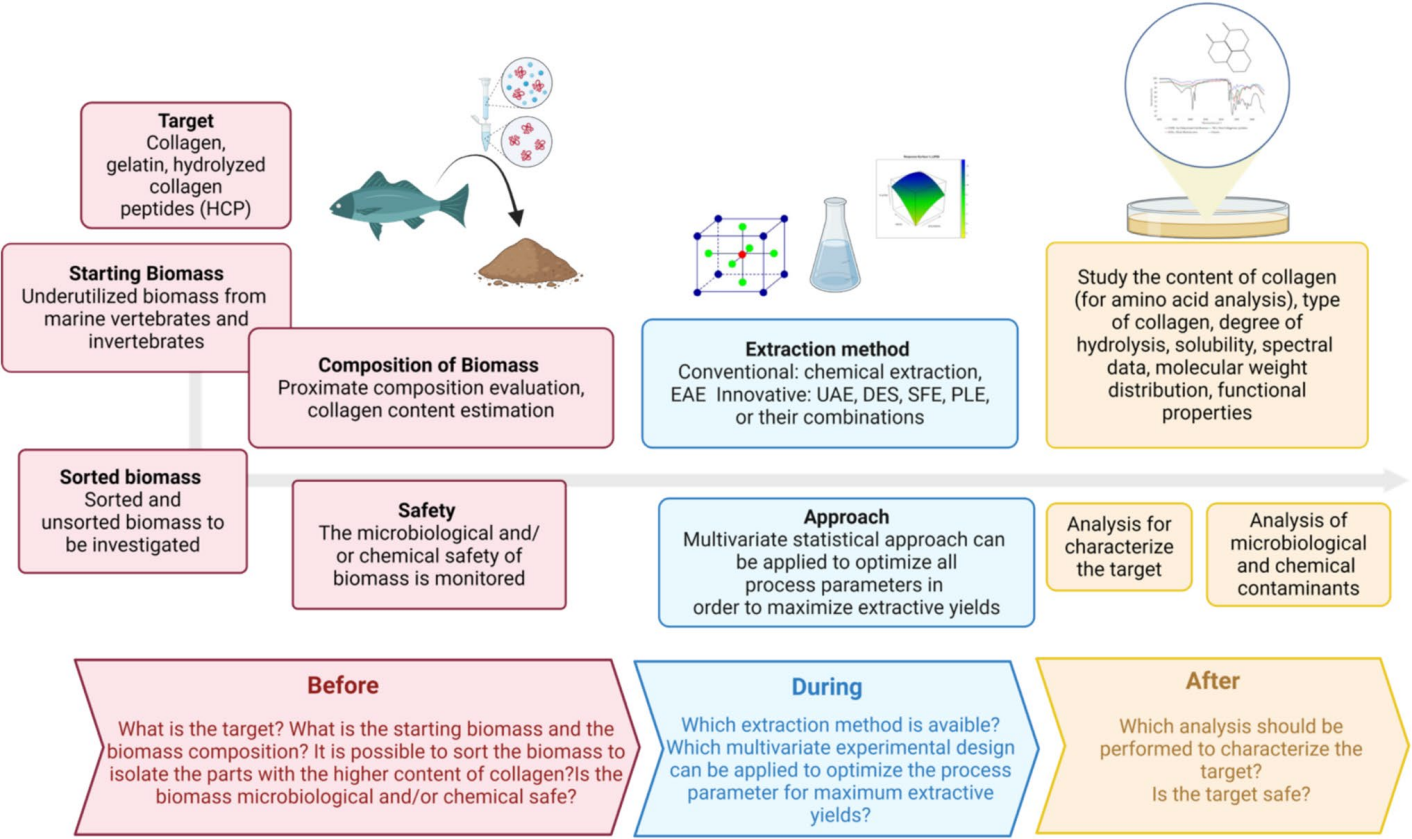
Scheme of the whole extraction process of collagen or HC peptides. The image was created using BioRender (https://www.biorender.com/). UAE, ultrasound extraction; DES, deep eutectic solvent extraction; SFE supercritical fluid extraction; PLE, pressurized liquid extraction; EAE enzyme-aided extraction

Generally, as reported in the literature, collagen extraction from fish sidestreams involves several steps: chemical pretreatment, extraction, precipitation, recovery, and formulation (such as freeze-drying or spray-drying) of the extracted collagenic fraction. In other words, starting from an organic biomass, it is possible to isolate a specific protein fraction, which can then be purified and stabilized, transforming it into a white to pale yellow powder. Nevertheless, it is important to highlight that these steps just represent the core of a larger process, which involves both previous feasibility evaluations and subsequent analytical characterizations. A scheme of the whole process is summarized in Fig. 6. As pointed out in the scheme, it is possible to divide the process into three main phases: (1) “before the process,” (2) “during the process,” and (3) “after the process.”

Regarding the initial “before phase,” it is important to highlight the type of bioactives (target compounds), such as collagen, gelatin, and HCP. In addition, the starting biomass will influence numerous of the process steps. Generally, it is customary to begin with precisely sorted biomass to minimize extraction efforts and, simultaneously, increase extraction yields. Depending on the type of marine source, specific parts or tissues can be isolated and exploited based on their collagen content and the feasibility of extracting it. Starting from unsorted biomass is much more complex, as reported in the literature (Orlandi et al. 2023; Grasso et al. 2023; Grasso et al. 2024a, b and Grasso et al. 2024a, b). Although it can be a challenging process, starting from unsorted biomass is sometimes worth exploring, as skipping the sorting phase can ensure a higher value of the final bioactives extracted. In any case, properly designing the preservation of fish sidestreams is crucial to maintaining high collagen quality. Adequate storage and quick processing are essential to prevent degradation and retain functional attributes. High-quality collagen derivatives can be obtained from the skins, bones, cartilages, scales, swim bladders, heads, and viscera if they are properly preserved, and the potential contaminants are precisely monitored. The “during phase” is the core of the entire process, involving chemical pretreatment (which varies based on the physical state and chemical composition), extraction, precipitation, recovery, and the final formulation of the extracted collagen fraction into a powder, typically through freeze-drying or spray-drying (Ali et al. 2018; Jafari et al. 2020). Depending on the specific marine sources, various techniques have been proposed for collagen extraction. These include traditional methods such as conventional chemical extraction and enzyme-aided extraction (EAE), as well as innovative approaches like ultrasound extraction (UAE), deep eutectic solvent (DES) extraction, supercritical fluid extraction (SFE), and pressurized liquid extraction (PLE). Sometimes, these methods are also used in combination (such as UAE and EAE) (Delikanlı Kıyak et al. 2024). Alternative extraction methods, including thermal or non-thermal treatments or the application of high temperature and pressure, enable high yields while reducing or eliminating energy consumption and the use of petroleum solvents. These methods also ensure a safe and high-quality extract (Turrini et al. 2018, 2019, 2021; Chemat et al. 2019). The properties and yield of extracted collagen are influenced by various factors, including the source and quality of the raw material, as well as the extraction conditions (such as extraction temperature, extraction time, solid/solvent ratio, and the type and concentration of chemical reagent and enzymes) (Laasri et al. 2023). All these parameters should be optimized using a multivariate experimental design to achieve the best compromise, aiming to maximize extraction yields (Leardi 2009). All extraction parameters for collagen are dedicated to both its separation and structural conservation; then, enzymatic treatment will be crucial for obtaining hydrolysates and defining their final characteristics (such as molecular weight, type of peptides, and degree of hydrolysis (DH)). During pretreatment, the raw material is washed, cleaned, separated, and ground or cut into small pieces to facilitate for the further extraction process (Gaikwad and Kim 2024). Pretreatment steps allow the removal of non-collagenous components such as lipids, non-collagenous proteins/peptides, and pigments and improve the collagen by using a weak acid or base to break down cross-linked collagen under controlled conditions (Gaikwad and Kim 2024). Demineralizing the raw fish biomasses is also necessary to enhance the final collagen yields. Ethylenediaminetetraacetic acid (EDTA) or hydrochloric acid (HCl) is recommended for demineralization purposes (Żelechowska et al. 2010; Duan et al. 2009). Collagen’s triple-helix fibers, stabilized by hydrogen bonds, make it insoluble in water, requiring chemical solvents, enzymes, or extraction instruments for effective solubilization and isolation. To ensure the integrity of collagen’s triple-helix structure and to avoid denaturation caused by higher temperatures, the entire extraction process should be maintained at 4 °C (Alam et al. 2022). Two widely used conventional methods are acid-solubilized collagen (ASC) extraction and enzyme-solubilized collagen (ESC) extraction. Acid-soluble collagen (ASC) extraction results in a traditional method for extracting collagen from fish. The primary goal of acid extraction is to disrupt the cross-links within the collagen helix, resulting in high-quality collagen. This process also leads to the depolymerization of high molecular weight proteins into shorter peptides, typically ranging from 0.3 to 8 kDa, as previously discussed. The ASC method, which involves various acid solutions, maximizes collagen yield and purity. The acid solution concentration, generally from 0.5 to 1 M, effectively allows the breakdown of both intra- and intermolecular cross-links while preserving the collagen triple chain structure. Acetic acid (AcOH) is commonly employed in extracting collagen from marine sources. Recently, Arumugam et al. (2018) found the highest collagen yield (1.93%) from fish skin with 0.5 M AcOH and 1.9 M NaCl, while Kuwahara (2021) obtained a 1.58% collagen yield from tilapia scales by passing CO_2_ through 0.1 M AcOH for 5 h. Although AcOH is commonly used in most studies, Tan and Chang (2018) recently investigated the use of various acids such as citric acid, HCl, and lactic acid. They found that the highest collagen recovery rate (64.19%) from minced catfish skins was achieved using HCl at pH 2.4 containing 23.6 KU/g pepsin. Bhuimbar et al. (2019) reported that lactic acid yielded the highest collagen extraction (45%) from black ruff fish skin. This was followed by formic acid (32%), tartaric acid (31%), citric acid (31%), and acetic acid (25%). In contrast, HCl and H_2_SO_4_ produced negligible yields. These results differ from earlier studies that reported lower yields with HCl compared to AcOH. Enzyme-soluble collagen (ESC) extraction is the leading method for obtaining collagen from marine sources and represents a green and environmentally friendly biotechnology-based process. Enzymes are typically used to isolate specific protein compounds, resulting in high yields. This method avoids the use of organic solvents and toxic chemicals, reduces waste, and shortens processing time. When the enzyme pepsin is added during the extraction process, the resulting collagen is called pepsin-soluble collagen (PSC). This method is highly effective because the protease cleaves telopeptide cross-linked regions without compromising the integrity of the triple helix, thus hydrolyzing some non-collagenous proteins and increasing collagen purity. Other enzymes, including Alcalase, Neutrase, Flavourzyme, Papain, Trypsin, and Protamex™, are widely used to extract collagen starting from fish sidestreams (Gaikwad and Kim 2024). When native collagen is denaturated, it produces three α-chains in a random coiled form, which can be observed when collagen is thermally treated above 40 °C. Once separated, these chains undergo hydrolysis by either chemical means (acidic or alkaline media) or enzymatic processes, resulting in the production of HC. HC is composed of small peptides whose solubility and functional activities, such as antioxidant and antimicrobial properties, depend on the type and degree of hydrolysis, as well as the enzymes used in the process. Alternative extraction methods, such as thermal treatment or the application of high temperature and pressure (such as using subcritical water), have been proposed. Recently, Orlandi et al. (2023) reported an extraction protocol for HC using both the whole *Mugil cephalus* L. fish, which are typically undersized or considered “unwanted catches,” and a mixed biomass of the same fish, consisting of unsorted skin, fins, and tails. This mixed biomass simulates the sidestreams of fish processing, such as those generated during fish filleting. For the first time, the authors introduced a scalable process for extracting valuable proteins (e.g., HC and non-collagenous peptides) from unsorted mixed tuna scraps that have been dehydrated using an industrial patented process (Grasso et al. 2023; Grasso et al. 2024a, b). This marks a significant innovation in the field, as it eliminates the need for the preliminary sorting step, which is both costly and time-consuming. Additionally, the process of sorting biomass is not always feasible for all industrial operations. Moreover, the dehydration process improves the logistics of handling this highly perishable biomass by reducing its volume and ensuring its microbiological stability. Currently, the recovery of various valuable compounds from seafood sidestreams is performed by innovative UAE: For instance, UAE is reported to be successfully applied for collagen extraction from fish sidestreams, with a reduction of processing time and increased yields (Al Khawli et al. 2019). Regarding DES extraction, it has proven to be an efficient and eco-friendly method for extracting collagen peptides from fish sidestreams with high extraction efficiency (Bai et al. 2017). In addition, other advanced technologies, such as SFE and PLE, are gaining greater importance in marine collagen extraction (Delikanlı Kıyak et al. 2024).

In the “after phase,” collagen derivatives characterization involves several mandatory analyses to understand the extracted target compound thoroughly. These include proximate analysis, amino acid composition, solubility at different pH levels, thermal stability, molecular weight distribution, collagen type, FT-IR spectrum, and functional properties (e.g., bulk density, foaming capacity). Additionally, microbiological and chemical contaminants analysis (e.g., dioxins and furans, polychlorinated biphenyls, heavy metals) is essential. The biological activity of the compound will be evaluated as described in “Collagen and Hydrolyzed Collagen in Bioengineering for Clinical Applications”.

## Good Manufacturing Practices (GMP)

Collagen, gelatin, and HC obtained by an extraction process can be considered raw materials to be used in cosmetic, nutraceutical, and packaging industries. Within the EcoeFISHent project, collagen and HC are used for topical purposes, as cosmetic ingredients, and for nutraceutical purposes, while gelatin is used to obtain plastic packaging. To allow the use of all these derivatives by industry, it is important to take into consideration both the regulatory frameworks currently in force and the management of the quality systems.

**Fig. 7:**
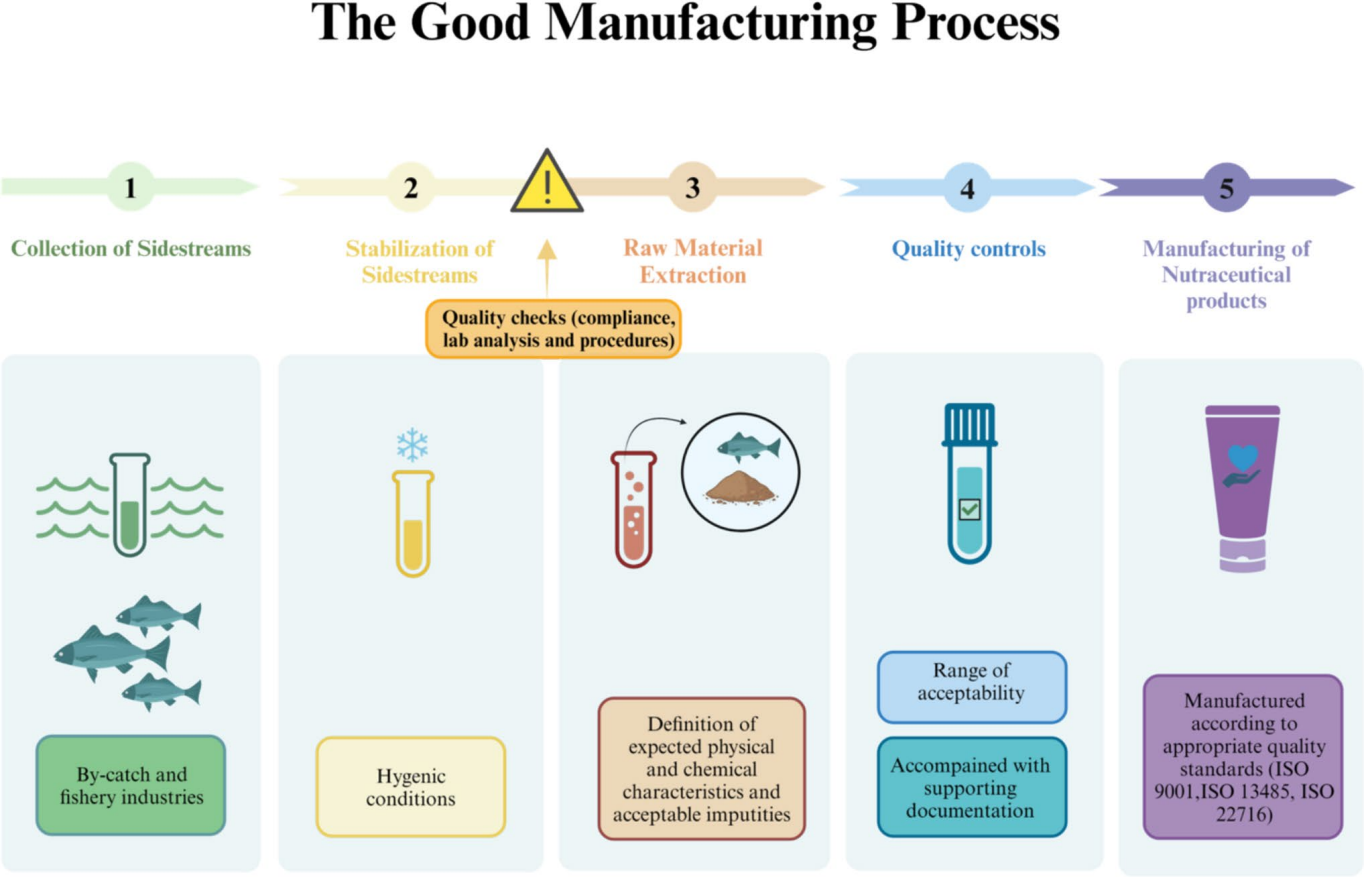
Scheme of the good manufacturing process in the EcoeFISHent project. The image was created using BioRender (https://www.biorender.com/)

The applicable regulatory frameworks depend on the legislation in force for the final products, such as Cosmetic Regulation 1223/2009, Food Regulations, and Food Contact Materials Regulation 1935/2004. In addition, these specific regulations include the requirement to manufacture according to good manufacturing practices (GMP). For example, the Cosmetic Regulation 1223/2009, article 8, specifically requires compliance with GMP in accordance with the relevant harmonized standards that, in this case, refer to the international standard ISO 22716. To help the industry verify and ensure that the quality standards are fulfilled for the entire supply chain, it is important to guarantee that also the raw materials used for each specific product are manufactured according to good manufacturing processes. For these reasons, specific practices for the extraction of collagen, gelatin, and HC are defined. To sum up, the EcoeFISHent project foreseen the production of cosmetic products and food supplements containing HC by food sidestreams (i.e., EcoeFISHent raw material) according to the usual manufacturing practices followed by the industry. The supply chain can be defined as follows (Fig. 7):

1. Collection of sidestreams;
2. Stabilization of sidestreams;
3. Raw material extraction;
4. Quality control;
5. Manufacturing of nutraceutical and cosmetic products.

Collection of sidestreams: the collection of sidestreams must follow procedures that guarantee the microbiological quality (see “Microbial Contaminants and Regulatory Limits in Food Grade Gelatin and Collagen Hydrolysates”) of biomass and minimize the oxidation as much as possible. In the case of transport, the cold chain must be guaranteed.

Stabilization of sidestreams: in the EcoeFISHent project, a patent technology provided by Themis S.p.A. can dehydrate and stabilize sidestreams used. Considering that the obtained raw materials will be included in food supplements, all the materials used in the machinery and the process must follow the indications required by food regulations. In addition, it is important to verify both the microbiological quality and chemical contaminants at the end of the dehydration process.

Raw material extraction: the specific required derivatives are obtained by enzymatic extraction (Grasso et al. 2023). The scale-up of the production process from the lab scale to the pilot scale must avoid the introduction of undesired solvents and must guarantee an advantageous yield and a physical and chemical characterization equivalent to what is defined at the lab scale.

Quality controls: quality standards are essential for deeming the extraction process acceptable and ensuring the reproducibility of the extracts. Three different aspects are to be considered:

- The physical and chemical characterization involves parameters such as the amino acid composition, MW distribution, and solubility. The selection of an acceptable range for these parameters depends on the intended final use of the extract, as they strongly impact its biological activity.
- The chemical contaminants considered depend on both the origin of the biomass and the intended final use. The threshold of acceptability depends on the most restrictive regulation related to the specific chemical.
- The microbiological quality has been extensively analyzed, as this is a critical parameter for the starting biomass and for meeting the regulatory requirements for nutraceutical products (that include both cosmetics and food supplements). This aspect is extensively described in “Use of Collagen in Nutraceuticals”.

Manufacturing of nutraceutical products: the manufacturing of nutraceutical products follows the regular quality system management required by the industry. All organized quality system management follow the guidelines provided by the standard ISO 9001. However, specific guidelines exist for different sectors. For example, the cosmetic industry follows ISO 22716 or Cosmetic Good Manufacturing practices.

## Microbial Contaminants and Regulatory Limits in Food Grade Gelatin and Collagen Hydrolysates

Hides, skins, and bones used as raw material for gelatin and HC production are typically heavily contaminated with microorganisms from soil and fecal matter. These organisms consist of potentially pathogenic and non-pathogenic vegetative cells and spores. The series of treatment steps involved in the production of gelatin and collagen from the selected raw materials have led to a significant reduction in the number of contaminating microorganisms. Indeed, the combined effects of exposure to high or low pH and heat treatment ensure that viable microorganisms are eliminated from the final product. However, similar to other food materials, gelatin can still be contaminated after manufacture (European Commission Health & Consumer Protection Directorate 2002). Therefore, it is crucial to prevent contamination during both use and production. The type of the organisms that can grow in gelatin solutions and gels are influenced by several factors, with pH being the most significant (Gelatin Manufacturers Institute of America 2019).

To prevent microbial contamination during manufacture, it is essential to monitor these parameters along with the time and temperature applied during the process steps. The use of hazard analysis and critical control points (HACCP) and good hygienic practice (GHP) is fundamental to produce foodstuffs. The HACCP system includes the determination of critical control points (CCP) and the establishment of critical limits (CL). CL must be established for pH, acid/base concentration, and treatment time and temperature at appropriate processing steps, which are considered CCPs for the safe production of gelatin and collagen hydrolysates. The Scientific Committee on Food of the European Commission finds that GHP and HACCP can be verified and validated microbiologically. The Committee proposed retaining *Salmonella* as a mandatory microbiological criterion to reflect the risk of the recontamination (European Commission Health & Consumer Protection Directorate 2002).

Most countries have microbiological specifications for gelatin and HC, but these requirements are generally not very stringent (Cole 2011). In the European context, the specific health conditions for gelatin intended for human consumption are outlined in Regulation (EC) No 853/2004 of the European Parliament and of the Council, Annex III, Section XIV. This regulation specifies the requirements for raw materials, including their transport and storage, the gelatin manufacturing processes, the quality standards for gelatin, and the packaging, storage, and transport of the final product (European Parliament and of the Council of 29 April 2004).

Concerning microbiological parameters, the maximum values for edible gelatin and collagen are specified in Regulation (EC) No 2073/2005, Annex I, Chapter 1, point 1.10. For food-grade gelatin, the only mandatory test required is for *Salmonella* (The Commission of the European Communities 2005)*.*

However, the Gelatin Manufacturers of Europe (GME), a key platform and primary reference in the gelatin industry, has proposed extending the bacteriological tests for edible gelatin and collagen peptides to include additional parameters. In the context of self-monitoring, food companies, especially those producing dietary supplements, can adhere to the parameters defined by the European Pharmacopoeia and its related limits. These parameters align with those of the GME, with the addition of molds and yeasts. Table 2 is a summarized table of the tests required or recommended to ensure the microbiological quality of gelatin (standardized methods for the testing of edible gelatin—gelatin monograph 2023).

**Table 2:** Tests required or recommended to ensure the microbiological quality of gelatin

Parameters	Food regulation EC/2073/2005	European Pharmacopoeia	GME requirements for edible gelatin
*Salmonella*	Absence/25 g (*n* = 5)	Absence/10 g	Absence/25 g
Total aerobic microbial count	/	max1000 CFU/g	< 1000 CFU/g
*Escherichia coli*	/	Absence/g	Absence/10 g
Anaerobic sulfite-reducing spore	/	/	< 10 CFU/g
Yeast and molds	/	max100 CFU/g	/

Some of these contaminants may be pathogenic for humans and could be a threat to human health. Furthermore, contaminants may exhibit gelatinase activity. Enzymatic degradation of gelatin would cause a reduction in the quality of gelatin and its derivatives without necessarily posing a danger to human health. To avoid quality issues in the final product, in addition to the previously defined parameters, further microbial contaminants can be assessed through self-monitoring (De Clerck et al. 2004).

For example, species belonging to the *Bacillus* genus or related genera are known to produce different proteases, including gelatinases (Zukowski and Mark 1992). Enzymatic degradation of gelatin would affect the viscosity and, therefore, the quality of the product itself and its derivatives. Moreover, because of this degradation, essential nutrients may become available for gelatinase-negative contaminants, promoting their growth. Furthermore, some of these contaminants may be pathogenic for human beings, which is of great concern, especially for the applications of gelatin in food and pharmaceutical products (De Clerck and De Vos 2002). There are also different species of *Clostridium*, *Staphylococcus*, and *Pseudomonas* that exhibited gelatinase activity, which indicated deteriorative effects on the quality of gelatin by enzyme gelatinase affecting the gelling capacity and viscosity of the gelatin. *Clostridium perfringens*, which is an anaerobic endospore former, is the causative agent of food poisoning gas gangrene in humans. *Staphylococcus aureus* is a pathogenic organism with food poisoning potential. *Pseudomonas aeruginosa* is also considered a potential pathogen in immunocompromised patients. Therefore, the detection of such microorganisms can both enhance the food safety of the final product and prevent its deterioration.

## Applications of Collagen

Collagen possesses a range of biochemical and biophysical properties that make it a crucial biomolecule. These attributes encompass solubility, strength, helping intracellular interactions, controllable stability, and biodegradability.

**Fig. 8:**
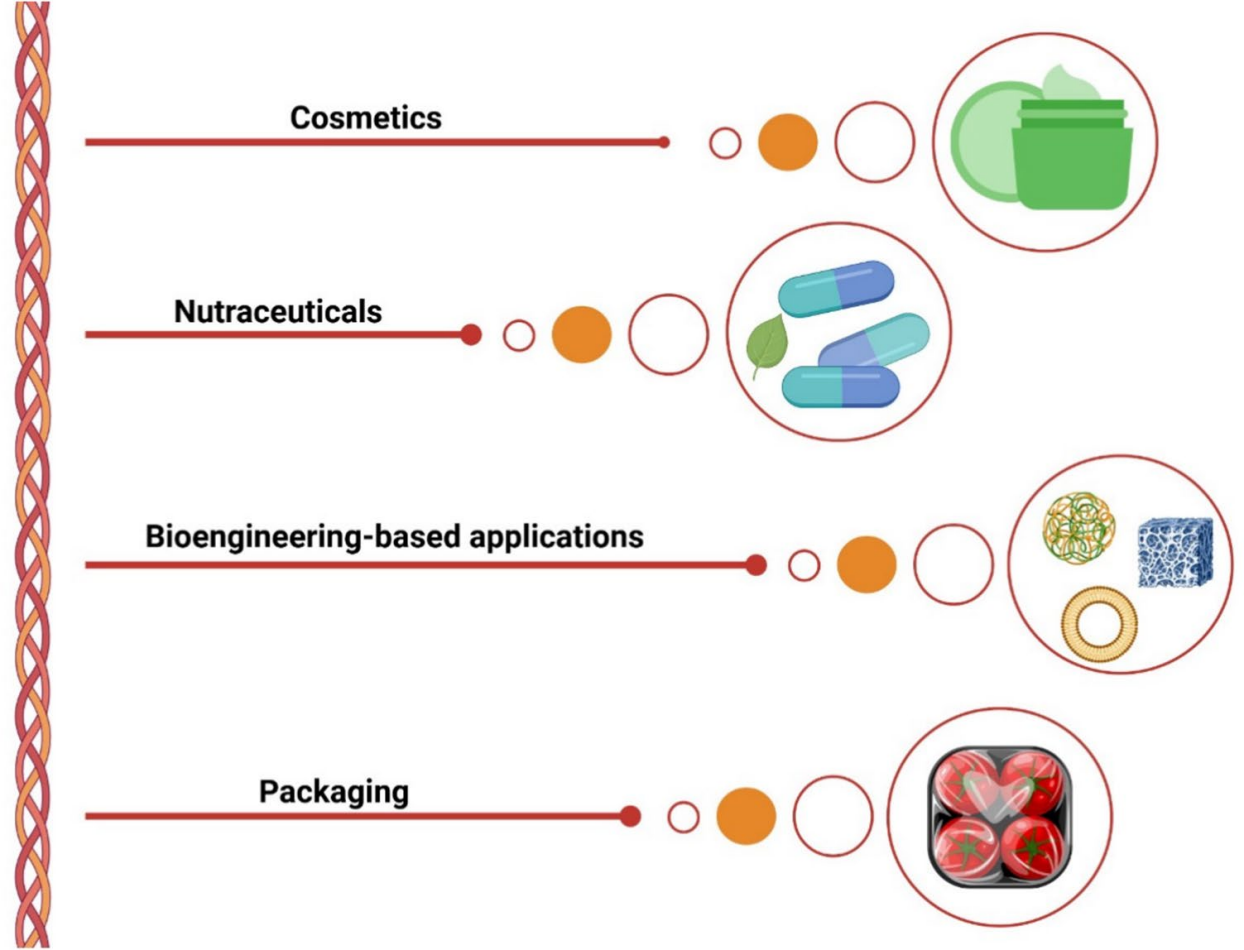
List of possible applications of collagen in several emerging sectors: cosmetics, nutraceutical, bioengineering-based, and packaging. The image was created using BioRender (https://www.biorender.com/)

A notable physical–mechanical property of collagen is its high tensile strength and minimal extensibility, contingent on the amount of insoluble collagen present (i.e., the number of cross-links) and its interaction with glycoproteins and proteoglycans. Consequently, collagen is proficient in transmitting forces of substantial magnitude, both tensile and compressive (Hulmes 1992). The chemical properties of collagen hinge on the presence of covalent cross-links, which confer controllable stability. These cross-links come in two varieties: intramolecular and intermolecular. Introducing cross-links into soluble collagen in vitro through physical or chemical reagents grants it structure and stability (Hulmes 1992). Then, collagen is widely accepted as one of the most versatile molecules playing a pivotal role in different sectors of the modern economy, i.e., nutraceuticals, cosmetics, biotechnology, and food packaging (Fig. 8).

### Use of Collagen in Cosmetics

Healthy skin is defined by the right structure and function of skin components. To maintain the skin in an attractive condition and reduce the aging process, its structures and functions must be supplemented and safeguarded. Human skin is composed of epidermis, dermis, and subcutaneous adipose tissue. In the dermis layer, collagen and elastin have a pivotal role to maintain the structure and elasticity of the skin (Baumann et al. 2021).

As human ages, collagen fibers thicken and shorten, reducing type I collagen and altering its ratio. Collagen and elastin density in the dermis decreases, making the skin thinner and less elastic, and hyaluronic acid levels drop, causing reduced hydration and wrinkles. Collagen synthesis declines, with a 1% annual loss after age 40 and a 75% decline by age 80 (Uitto 1997; Lephart and Naftolin 2021). Furthermore, both endogenous (hormones, genetic heritage, alterations of cellular metabolic processes) and exogenous (pollution, UV radiation, smoking) factors influence the appearance of the skin, in particular, by damaging collagen and elastin and, above all, increasing the risk of skin diseases (Rispo et al. 2024; Farage et al. 2008; Shields et al. 2012; Sibilla et al. 2015; Varani et al. 2006). For these reasons, collagen represents one of the most important ingredients for cosmetic formulations thanks to the moisturizing, regenerating, and film-forming properties. Its versatility and ability to be used in relatively low concentrations but with significant impacts on the finished products make it a valuable component (Avila Rodríguez et al. 2018).

Water is a crucial element since hydration plays a fundamental role in maintaining the skin health. Typically, cosmetic products that are applied topically are oriented to maintain and improve skin hydration. Indeed, variations in skin water content play a key role in defining its hydration level. Collagen exhibits a good water retention ability because it can bind water, maintaining its proper content in the skin during the day and making the skin moisturized and softened. The entire collagen molecule is too big to soak into the skin (Peng et al. 2004), but they remain on the surface working as water absorbers through hydration and as protectors against microbial infiltration in cases of wounded tissue (Chattopadhyay and Raines 2014). On the other hand, HC, composed by short polypeptides, can penetrate the deepest layers of the skin, where it can directly contribute to maintain and improve skin hydration. This is due to the presence of many natural moisturizing factors (NMFs), such as Ser, Asp, Hyl, and Hyp.

Water skin content plays also a crucial role in wrinkles formation and reduction of elasticity (Verdier‐Sévrain and Bonté 2007). The particularity in the skin aging is that in young skin, a significant portion of water is bound to collagen, resulting in a well hydrated, flexible, and elastic skin. However, water can be found in free form in aged skin causing water molecules to bond together instead (Aguirre-Cruz et al. 2020; Sklenářová et al. 2022; Ahmed et al. 2020). Therefore, collagen and its derivatives are especially used in the cosmetic field to mitigate skin wrinkles.

Living in an oxidant atmosphere and performing oxidative metabolism, human tissues, including skin, are constantly exposed to reactive oxygen species (ROS) as a source of oxidative stress, inducing damage in merely all the biological macromolecules such as proteins, nucleotides, and lipids, thus ultimately causing cell or tissue damage (Cai et al. 2015). In the skin, ROS are recognized to contribute to the skin aging. In this context, HC possesses a well-documented antioxidant activity that can vary depending on the molecular weight of HC and on the source of collagen from different terrestrial or marine species that influences amino acid composition and sequence (Li et al. 2022).

The skin is constantly exposed to the sun’s UV rays, which can lead to a peculiar aging process known as photoaging. UV-exposed skin is subjected to increased ROS, decreased skin hydration, and other mechanisms reviewed in Li et al. (2022). HC administered to UVB-damaged skin showed some benefits in retaining moisture, enhancing antioxidant activity, and promoting collagen and elastin protein production (Fowler 2012). Besides the presence of NMFs and antioxidant properties, HC can also decrease excessive melanin formation, which is responsible for ROS production. The underlying mechanism of action is not fully understood and may be due to some direct actions on tyrosinase activity by combining tyrosine residues in collagen peptides with the active part of the enzyme. Moreover, the decrease in melanin formation driven by collagen is also attributed to the indirect modulation of AMPc and/or MAPK pathways.

Researchers have also demonstrated that collagen and HC are able to accelerate wound healing by helping tissue to regenerate (Secchi 2008). This is an important role because the skin serves as a major protective barrier, preventing both desiccation and mechanical, chemical, and thermal harm to internal tissues (Takeo et al. 2015).

Both collagen and HC are present in a plethora of cosmetic products with many applications. Generally, products containing collagen as an active ingredient could be divided into fillers, oral products, and products used on the skin (gels, creams, serums, and masks) (Jadach et al. 2024). The application of collagen as a filler offers some advantages in aesthetic medicine thanks to its low invasiveness and its ability to increase skin smoothness while decreasing wrinkles (Cohen et al. 2006).

Regarding oral products (extensively described in paragraph 7.2), these collagen preparations were primarily designed for individuals dealing with connective tissue diseases, particularly those affecting cartilage and joints (Asserin et al. 2015). As a result of consuming HC, these peptides are found in the bloodstream, generating chemotactic properties for skin fibroblasts and supporting their reconstruction processes (Sionkowska et al. 2020). In this way, supplementation with collagen hydrolysates may delay the changes in the extracellular matrix that occur with aging (Bauman 2004; Alves et al. 2017; Jafari et al. 2020; Rodríguez et al. 2017). Additionally, the hydrophilicity makes HC more easily introducible into cosmetic formulations. To this aim, the gel is a form of preparation very often used in dermatological treatments and cosmetics due to its ease of application, high water content (95–99%), and its ability to create a film on the skin surface (Ciszek 2017) The other forms of cosmetic products with collagen are creams, serum, or masks. They are composed of water-in-oil or oil-in-water emulsions with higher or lower concentration of collagen or HC, depending on their use (night or day) or on their category (cream or serum).

In conclusion, the use of collagen in cosmetics offers a wide range of possibilities. Depending on the formulations and molecular weight, collagen exhibits a plethora of beneficial effects, making it a versatile and valuable ingredient with several cosmetic applications (Alves et al. 2017; Van Der Rest and Garrone 1991b; Gómez-Guillén et al. 2002; Rawdkuen et al. 2013).

### Use of Collagen in Nutraceuticals

Nutraceuticals are products that are used at the intersection of nutrition and pharmacy. The term was first used by Dr. Stephen DeFelice in 1989, who combined the two words “nutrition” and “pharmaceutical.” Since then, nutraceuticals have become a highly researched topic. DeFelice defined nutraceuticals as “any substance that is a food or part of a food and provides medical or health benefits, including the prevention and treatment of disease” (DeFelice 1995). The purpose of nutraceutical products is to help maintain optimal health through preventive care and to provide additional therapeutic effects along with nutritional benefits. Nutraceuticals can help prevent various diseases, including cardiovascular and skin conditions. Incorporating effective nutraceuticals into the daily diet could potentially delay or prevent the onset of pathological conditions and, in that sense, reduce the need for pharmaceuticals (Santini and Novellino 2018). Many terms have been introduced surrounding food and food-related products with health benefits, such as “dietary supplements,” “food supplements,” “functional foods,” and “pharma food.” The term “nutraceutical” currently lacks a clear definition (Puri et al. 2022; Santini and Novellino 2018; Teoh et al. 2019). Attempts have been made to differentiate “nutraceuticals” from “food supplements.” The former was defined as products designed for the prevention and/or treatment of a pathological disease (with an identified epidemiological target), supported by strong scientific evidence of pharmacological effect, safety, efficacy, and a defined mechanism of action. In this attempt, food supplements are defined as products used to treat micro- or macronutrient deficiencies, such as iron deficiency (Santini and Novellino 2018). A study on factors influencing consumers’ decisions to use nutraceuticals revealed that the key motivating factors for using these products were perceived health benefits and safety, as well as advice from healthcare professionals, friends, and family. The barriers for using nutraceutical products were a lack of belief in the health benefits, high cost, and lack of knowledge among the consumers (Nasri et al. 2014).

The skin is our largest organ, and nutraceuticals can be used to delay the aging process and improve its health (Dini and Laneri 2019).

The market for nutritional supplements promoting the health of skin, nails, and hair is growing, and this has yielded new terms such as “nutricosmetics.” Nutricosmetics involves different health-promoting products that are ingested and believed to positively affect human skin, hair, and nails. Such products are often branded to “increase beauty from within.” Common examples are collagen peptides, omega-3 fatty acids, and hyaluronic acid (Ruocco et al. 2016). Collagen and collagen peptides are often used in nutraceutical formulations for skin treatments. Formulations containing collagen have been found to reduce wrinkles and positively affect the skin matrix synthesis. Several clinical studies have been performed on the effects of collagen peptide ingestion. Most studies focus on skin-related conditions, e.g., wrinkles, pores, and pigmentation (Ruocco et al. 2016). However, some also focus on other areas, such as pain, mental and physical health, and joint and bone diseases.

In a study to assess the effect of nutraceutical collagen peptides from bovine and porcine sources, enhanced cellular proliferation and migration resulted in improved wound closure of young and aged fibroblasts and keratinocytes (Mistry et al. 2021). Following the ingestion of 10 g of porcine collagen peptides, the peak concentration of collagen (as measured by total Hyp concentration) was observed 2 h after ingestion, at levels equivalent to those tested in the in vitro experiments. In a study performed on 34 healthy Indian women, the ingestion of a collagen-antioxidant mixture (containing 5 g of fish collagen peptides) reduced wrinkle width, open pores, skin roughness, and the color of hyperpigmented blemishes. Skin hydration, firmness, and barrier function were also improved after 30 and 60 days of treatment (Motwani et al. 2020). A clinical study observed improvement in skin hydration when 10 g of Peptan from fish (Peptan®F) or porcine Peptan (Peptan®P) was consumed (both Peptan products were provided by Rousselot, Ghent, Belgium). The moisture levels were significantly improved after 8 weeks of treatment compared to the placebo group. The effect was more pronounced when Peptan®P was used, rather than Peptan®F. Treatment using Peptan®F also resulted in increased collagen density and reduced collagen fragmentation in the dermis (Asserin et al. 2015). VERISOL® (GELITA AG, Eberbach, Germany) is a commercially available collagen peptide supplement that has been studied for its effects on skin, hair, and nails. Clinical studies have shown improved skin elasticity and reduced wrinkle volume, increased procollagen type I and elastin content, improved nail health and hair thickness, and reduced cellulite and skin waviness after ingestion of VERISOL® (Schunck et al. 2015; Proksch et al. 2014; Rawdkuen et al. 2013).

### Collagen and Hydrolyzed Collagen in Bioengineering for Clinical Applications

Collagen and its hydrolyzed form have revolutionized bioengineering due to their diverse functionalities and remarkable properties in clinics. In living beings, collagen provides structural support and strength to various tissues including skin, bones, tendons, and cartilage. As deeply described before, its triple-helix structure endows it with high tensile strength, making it a critical component in maintaining the integrity and elasticity of connective tissues. Collagen could be extracted by a plethora of animal sources and considered its biocompatibility, biodegradability, water solubility, easy extractability, minimal risk of transmitting zoonoses, and low immunogenicity; marine collagen plays a pivotal role in the biomedical field (Jafari et al. 2020).

In the bioengineering sector, marine collagen and its hydrolyzed derivatives are more and more getting the attention of the scientific community, addressing the need to improve bioengineered devices, including biomaterials, carriers for drug delivery, and bio-based sensors. Marine collagen and derivatives have been extensively used in wound healing applications due to their ability to promote cell adhesion, migration, and proliferation, which are essential steps for tissue repair and regeneration (Cruz et al. 2021). Collagen dressings, for instance, provide a moist environment that facilitates faster healing and reduces scarring. In the tissue engineering panorama, marine collagen-based scaffolds are employed to create three-dimensional structures that mimic the extracellular matrix, supporting the growth and differentiation of cells into functional tissues (Liu et al. 2022). Collagen-based scaffolds offer good mechanical properties, providing the necessary support for the developing tissue. This is crucial in load-bearing applications, such as bone and cartilage engineering (Elango et al. 2018 and Mredha et al. 2017) where the scaffold must withstand mechanical stresses while supporting new tissue growth. Furthermore, collagen scaffolds are biodegradable, breaking down into harmless by-products easily resorbed by the body, ensuring the scaffold gradually disappears as the new tissue forms, and leaving no immunogenic molecules. On the other side, HC, made of smaller peptides, is more readily absorbed and utilized by cells compared to native collagen, enhancing its effectiveness in promoting tissue repair and regeneration. It is considered a good candidate to create injectable hydrogels that serve as scaffolds for cell delivery, filling irregularly shaped defects and providing a supportive environment for cells to grow and differentiate, particularly useful in minimally invasive procedures for tissue repair (Li et al. 2024). Marine collagen is often combined with other biopolymers and growth factors to create composite materials with enhanced properties, providing better mechanical strength, controlled degradation rates, and improved biological activity, making them suitable for various tissue engineering applications (Chandika et al. 2021). Collagen-based scaffolds are extensively used in skin tissue engineering to treat wounds, burns, and ulcers, providing a suitable environment for keratinocytes and fibroblasts to proliferate and form new skin tissue (Lim et al. 2019b). In bone repair, collagen and HC scaffolds support the differentiation of mesenchymal stem cells into osteoblasts, promoting the formation of bone. Fish-derived collagen has also been studied for cartilage regeneration (Pallela et al. 2013). For cardiac tissue engineering, collagen scaffolds are employed to create constructs aiming to repair damaged heart tissue by supporting the growth and organization of both cardiomyocytes and endothelial cells, contributing to the regeneration of functional cardiac tissue. In vascular tissue engineering, collagen from jellyfish and poly(lactic-*co*-glycolic acid) were used to fabricate vascular graft by freeze-drying and electrospinning processes (In Jeong et al. 2007). In nerve regeneration, collagen conduits guide the regrowth of injured peripheral nerves, providing physical support and biochemical signals that promote the migration and alignment of Schwann cells (Chen et al. 2020). In drug delivery, collagen is used to create matrices that encapsulate drugs and release them gradually over time (Nicklas et al. 2009). This controlled release is particularly beneficial for medications that require sustained therapeutic levels, minimizing the need for frequent dosing and enhancing patient compliance. Marine collagen-based carriers have been studied for protein delivery (Calejo et al. 2012). Additionally, collagen hydrogels and sponges are commonly employed as drug delivery systems, as they can absorb large amounts of drugs and release them at the site of application, making them particularly useful in localized drug delivery, such as in wound healing or, in the form of gelatin, in tumor treatment (Kang et al. 2019).

Marine collagen can also be formulated into microparticles (Swatschek et al. 2002) for topic application in the pharmaceutical and cosmetic field. In wound healing, collagen and HC dressings can be impregnated with antibiotics (Veeruraj et al. 2012) or loaded with amino acids (Langasco et al. 2017) or bioactive compounds (Siaghi et al. 2024) to enhance the regeneration process. These dressings provide a good environment that promotes healing and protects the wound from infection while delivering therapeutic agents directly to the wound site.

In terms of biosensor development, marine collagen’s natural properties (safety, thermostability, interconnected porosity, high denaturation temperature, and biocompatibility) stand out, making it particularly suitable for medical diagnostics and in vivo applications (Bedi et al. 2022). This biocompatibility ensures seamless integration with biological tissues and fluids, minimizing adverse reactions and enhancing sensor accuracy. Marine collagen is an excellent immobilization matrix for enzymes, antibodies, and other biomolecules, forming stable gels and films that maintain biological activity. This capability is crucial for biosensors that depend on specific biochemical interactions to detect target analytes (Srivastava et al. 2015).

Marine collagen-based biosensors could be extensively used in medical diagnostics for detecting biomarkers in bodily fluids. For instance, a 3D porous and biostable collagen-based scaffold has been studied to improve the biocompatibility of implantable glucose sensors, enhancing the angiogenesis in the surrounding area (Ju et al. 2008). Another example is represented by collagen-based biosensors for the rapid detection of viable cells of pathogenic *Listeria monocytogenes*, the toxin listeriolysin O, and the enterotoxin from *Bacillus* species (Banerjee et al. 2008). Recently, Derkus et al. (2016) developed an innovative aptasensor for the detection of thrombin levels in the blood and cerebrospinal fluid obtained from patients with multiple sclerosis, myasthenia gravis, epilepsy, Parkinson’s disease, and polyneuropathy.

Advances in tissue engineering, nanotechnology, and biosensors are expected to yield next-generation marine collagen-based medical devices with improved properties. As our understanding of the interactions between collagen-based materials and biological systems deepens, the potential for creating innovative and highly effective tools using collagen and HC will continue to grow, driving advancements in healthcare, environmental protection, and food safety.

### Use of Collagen and Its Derivatives for Packaging

Currently, there are two main approaches to address the environmental issues caused by plastic packaging. The first approach focuses on increasing recyclability and recycling rates of fossil-based polymers, such as polyethylene, polypropylene, polyethylene terephthalate, and polystyrene. The second approach involves the use of biodegradable polymers. Polymers derived from fish waste are promising alternatives to synthetic polymers due to the fact that they are bio-based and biodegradable. Thanks to its good film-forming properties, biocompatibility, and biodegradability, fish gelatin has been studied recently for the preparation of biodegradable films in food packaging applications, substituting conventional non-biodegradable polymers and other mammalian-based gelatin (Lionetto and Esposito Corcione 2021). Food packaging is designed to preserve and protect all foods, mainly from oxidation and microbial contamination, thus improving their shelf-life (de la Caba et al. 2019).

Food sources of lipids that are highly unsaturated, such as fish and seafood, can undergo oxidation that can deteriorate food quality, with consequences such as off-odors, off-flavors, nutrition losses, and color change. Thus, a film or coating with a high oxygen barrier is important to control oxygen exchange between food and surrounding atmosphere, postponing its degradation (de la Caba et al. 2019).

Gelatin cannot be processed like common thermoplastic polymers due to its strong intermolecular and intramolecular hydrogen bonds. Demonstrated the production of fish gelatin-based edible films by combining the use of a twin-screw extruder and a compression molding (Krishna et al. 2012). Many studies report the manufacturing of gelatin films by casting method using an aqueous solution (Luo et al. 2022). This method is based on the dispersion or solubilization of gelatin, with plasticizers or additives, in a solvent medium, followed by the solvent removal to obtain the film. In the presence of appropriate plasticizers, gelatin can melt and flow if treated at a temperature in the range of 60–160 °C and under shear. Indeed, in most cases, the manufacturing of gelatin-based products requires the use of plasticizers. Typically, plasticizers are low molecular weight compounds compatible with gelatin and are usually polar and hydrophilic. The ability of the plasticizer to interact with gelatin is fundamental, and its efficiency depends on several factors, including molecular weight, shape, and functionality. Many studies (Hoque et al. 2011; Kaewprachu et al. 2018; Azmi et al. 2020; Riquelme et al. 2015) report the use of glycerol and other types of plasticizers, such as sorbitol or glucose, incorporated into biopolymer film-forming solutions to increase the flexibility and processability of resulting films, for instance, compared the effects of three different plasticizers, glycerol, sorbitol, and polyethylene glycol (PEG), on the properties of fish protein-based film. Higher elongation at break and water vapor permeability (WVP) were found in glycerol-plasticized films, whereas sorbitol, having less ability to bind water, showed lower WVP9 (Kaewprachu et al. 2018). Investigated the effect of PEG as a plasticizer, with different molecular weights and showed that lower molecular weight PEG gives a better plasticizing effect and lower WVP (Cao et al. 2009).

The physical properties of gelatin depend on molecular weight distribution and amino acid composition and thus influence the mechanical and barrier properties of the resulting films. If a low molecular weight portion predominates in a gelatin solution, more flexible films are obtained, which may be due to protein heat degradation during the extraction or evaporation step (Gómez-Guillén et al. 2009, 2011). Muyonga et al. and Carvalho et al. observed that the gelatin-based film, with a higher fraction of low molecular weight peptides, had lower tensile strength and higher percentage elongation (Muyonga et al. 2004; Carvalho et al. 2008).

Gelatin in food packaging can be used both as bulk material in a flexible film and as a coating of a plastic film (Said and Sarbon 2022; Ramos et al. 2016). The main advantage is related to the reduction of oxygen transmission rate, with clear benefits for the food preservation. Ciannamea and colleagues compared the oxygen permeability of gelatin-based film and other films from synthetic polymers. Their results suggest the potential use of gelatin-based films to manage the oxygen exchange between food and the surrounding atmosphere. Ciannamea et al. (2018) reported a study on the blend of gelatin and sodium-caseinate solution to obtain films with low oxygen transmittance rate and at the same time lower humidity dependence than the one of neat gelatin films (Choi et al. 2023). Moreover, an elderberry extract was incorporated into the blend to improve the antioxidant ability of the films. The film obtained in their study can be used as active packaging to maintain food quality and increase shelf-life. Debeaufort et al. studied the properties of polylactic acid (PLA) film coated with a cross-linked suspension of plasticized gelatin incorporating phenolic compounds. As a result, gelatin-based coating allowed to reduce by several orders of magnitude the oxygen permeability of PLA films in low relative humidity conditions (Debeaufort et al. 2022).

Despite the good performance in terms of oxygen barrier properties, the use of fish gelatin films in food packaging is limited by its hygroscopic nature, because it tends to swell when in contact with foodstuffs with high moisture content. This is responsible for a drastic reduction in oxygen barrier and mechanical strength. One strategy to overcome this weakness is to associate the gelatin-based film with a moisture-resistant biodegradable polymer through laminating. Martucci et al. focused on developing a new biodegradable three-layer film based on modified gelatin layers produced by compression molding. Multilayer film was composed of two outer moisture and mechanical-resistant layers made of dialdehyde starch-cross-linked gelatin film plasticized with 30% glycerol and a blend of non-toxic sodium montmorillonite with glycerol-plasticized gelatin as an inner layer. This multilayer film showed better moisture resistance and oxygen barrier properties than individual components and quite good mechanical properties (Martucci and Ruseckaite 2009). Moreover, it was studied the biodegradability in natural environments or landfills of dialdehyde starch-cross-linked gelatin multilayer.

Mechanical and thermal properties of fish gelatin for food packaging are based on cross-linking, thus contributing to improve the barrier capacity. Bio-based cross-linking agents have involved more attention to consider environmental and health concerns. Bhat and Karim investigate the cross-linking effect of different sugars (ribose and lactose) followed by UV radiation on the properties of fish gelatin films. In general, the tensile strength of the fish gelatin films increased after their exposure to UV radiation, but the elongation at break decreased notably. Regarding the sugar effect, the added ribose has led to a film with higher tensile strength than lactose (Bhat and Karim 2014). Recently, Maroufi et al. demonstrated the chemical cross-linking of fish gelatin with the aldehyde groups of *κ*-carrageenan (DAK-car) to improve the properties of gelatin film (Yavari Maroufi et al. 2021). DAK-car cross-linked gelatin-based film showed better value for tensile strength and elongation at break than simple gelatin film.

## Environmental Impact of Collagen Production: Life Cycle Assessment Perspective

The scientific literature at this time is very poor in terms of studies that examine the environmental impacts associated with the production of collagen and/or gelatin. The reference methodology for this type of analysis is certainly that of the life cycle assessment (LCA) (International Organization for Standardization: Geneva, Switzerland 2006) (Hauschild et al. 2018), which by examining all the life phases of a product tries to objectively evaluate—according to standards and internationally recognized models—the environmental impacts that are generated along a process, in relation to various environmental indicators.

A study of 2021 presents a life cycle assessment of collagen extracted from codfish skin at a laboratory scale (Bisht et al. 2021). The extraction process involved initially thawing and cleaning the frozen codfish skins manually. The skins were then segmented into smaller pieces, followed by the removal of non-collagenous elements by soaking the skins with 0.1 M sodium hydroxide (NaOH). Then, the skins were defatted using a 10% butyl alcohol solution. Collagen extraction utilized a 0.5 M solution of AcOH and DES. Collagen precipitates were obtained by adding NaCl and performing centrifugation. At the end, collagen was dried by freeze-drying.

The study evaluated three different extraction methods with three different solvents: AcOH, a mixture of urea and lactic acid (U:LA) at a 1:2 ratio, and a mixture of urea and propanoic acid (U:PA) also at a 1:2 ratio. The functional unit defined for the LCA was 1 g of collagen. The LCA results obtained per gram of collagen produced to facilitate comparisons of the environmental performance of the three solvent scenarios as shown in Table 3. The analysis highlights that using AcOH in collagen extraction results in the highest environmental impacts. Alternatives like U:LA (1:2) and U:PA (1:2) ratios in the solvent mix reduce these impacts by 10 to 15% across all categories. It must be mentioned that the extraction yields (weight of dried collagen per weight of dried skins) are quite similar in the three methods tested: 4.2%, 5.2%, and 5% for AcOH, U:LA, and U:PA, respectively.

**Table 3:** Impact categories results of all the three extraction methods: *AcOH* acetic acid, *U:LA* urea and lactic acid, *U:PA* urea and propanoic acid

Impact categories and unit of measurements	AcOH	U:LA	U:PA
Global warming (kg CO_2_ eq)	1.18	1.03	1.00
Ozone formation, human health (g NO_x_ eq)	2.53	2.15	2.13
Terrestrial acidification (g SO_2_ eq)	4.65	3.94	3.90
Mineral resource scarcity (g Cu eq)	0.111	0.107	0.100
Fossil resource scarcity (kg oil eq)	0.490	0.423	0.420

The extraction and purification stages of collagen preparation are recognized as having the most substantial environmental impacts, accounting for over 50% of the total impacts across all three methods evaluated. Specifically, when using AcOH as the solvent, the extraction and purification processes contribute approximately 70% to the environmental impacts, followed by the preparation of codfish skin, which accounts for about 25% of the impacts. A significant portion of the total impacts of around 70% is attributed to electricity consumption during mechanical stirring, centrifugation, and freeze-drying. It should be noted that these findings represent potential worst-case scenarios for the electricity consumption estimation since the calculations are based on the equipment’s nominal power rather than actual measurements (Bisht et al. 2021). The authors of the study recommended that changing the electricity mix by including more renewable sources such as photovoltaic or wind turbines could substantially reduce the environmental impacts of these processes by 64–69% (Bisht et al. 2021).

A 2020 study by Andonegi offers partial insights into the environmental impacts associated with collagen derived from bovine skin. Although our review focuses specifically on fish-based collagen, this study is relevant as a reference point due to similarities in the collagen extraction methods utilized. Moreover, no other studies have conducted an environmental analysis of collagen scaffolds derived from bovine skins, indicating that this subject has not gained significant attention (Andonegi et al. 2020a). In the same study by Andonegi, collagen scaffolds were prepared in the laboratory. The collagen extraction method employed was broadly similar to that detailed in the previously mentioned research, with the primary distinction being the incorporation of zinc oxide nanoparticles (ZnO NPs) to improve the properties of the collagen films, thus enhancing their suitability for biomedical applications (Andonegi et al. 2020b). The pretreatment involved using 1 M NaOH, and the extraction was carried out solely with 0.5 M AcOH, accompanied by mechanical stirring for 3 h. This study did not consider other solvents. Finally, the collagen samples were freeze-dried to eliminate moisture. The functional unit was defined as 5 g of collagen extracted from bovine skin. It must be clarified that this study does not report any details of how the system for LCA analysis was modeled and in particular which proxies were used for the various materials, as well as what the collagen yield is, compared to the starting material (bovine skin). However, some results are interesting as reported in Table 4, significant contributions to the environmental impacts during the collagen extraction process are made by terrestrial ecotoxicity, global warming, and ionizing radiation. Furthermore, freeze-drying and mechanical stirring account for 80% of the total environmental impacts, with electricity being the predominant source of these impacts. Since the electricity utilized in the process contributes significantly to the environmental impacts, the study suggests that optimizing these processes is essential to decrease the environmental burden related to the production of collagen scaffolds (Piccinno et al. 2016). Given that these scaffolds were originally produced on a laboratory scale, expanding these processes could help achieve significant reductions in the environmental impacts described earlier.

**Table 4:** Impact results of 5 g of collagen scaffolds

Impact categories	Unit	Values
Global warming	kg CO_2_ eq	0.7730
Stratospheric ozone depletion	kg CFC11 eq	4.09 × 10^−7^
Ionizing radiation	kBq Co-60 eq	0.4080
Ozone formation, human health	kg NO_x_ eq	0.0026
Fine particulate matter formation	kg PM_2.5_ eq	0.0019
Ozone formation, terrestrial ecosystem	kg NO_x_ eq	0.0027
Terrestrial acidification	kg SO_2_ eq	0.0048
Freshwater eutrophication	kg P eq	0.0003
Marine eutrophication	kg N eq	3.32 × 10^−5^
Terrestrial ecotoxicity	kg 1,4-DCB	0.8840
Freshwater ecotoxicity	kg 1,4-DCB	0.0105
Marine ecotoxicity	kg 1,4-DCB	0.0146
Human carcinogenic toxicity	kg 1,4-DCB	0.0233
Human non-carcinogenic toxicity	kg 1,4-DCB	0.3050
Land use	m^2^a crop eq	0.0211
Mineral resource scarcity	kg Cu eq	0.0007
Fossil resource scarcity	kg oil eq	0.2350
Water consumption	m^3^	0.0086

The two studies show a marked variability of the results with impacts on collagen production ranging (in the case of the global warming category) from a minimum of 0.15 kg CO_2_ eq/g of collagen (Andonegi et al. 2020a study) to a maximum of 1.18 g CO_2_ eq/g of collagen (work of Bisht et al. 2021). It should be noted that both studies examined the same system boundaries: starting from the pretreatment of the “raw materials” (i.e., fish and bovine skin, whose environmental burdens are excluded from quantification, similarly to what is done in a cutoff approach (Shen et al., 2010)) and till the production of the finished product. However, in the work starting from bovine skin, there is no reference to the process yield that would allow for a more appropriate weighting of the final results. Although the literature is very poor in providing benchmark analyses for estimating the environmental impacts of collagen, it is quite obvious that energy consumption is the main cause of the impacts in both the analyzed studies. As stated, processes carried out at laboratory scale are often characterized by high energy consumption for each phase. In general, scaling up the process allows to drastically improve energy efficiency and therefore reduce the overall environmental impact. At present, however, the literature does not report quantitative evaluations of environmental impact conducted for collagen extraction on a larger scale, and this is clearly a scientific gap that deserves further investigation.

It should be noted that collagen extraction from sources currently classified as waste (e.g., marine sidestream) offers the possibility of promoting circular economy practices. Through the valorization of classic waste flows, management and valorization opportunities can be highlighted for the fish sector and the entire value chain (Ruiz-Salmón et al. 2021). The analysis of environmental, economic, and social impacts can be the tool to highlight the benefit of these practices and to enable new production paths.

Environmental impact, enriched with economic and social analyses, relating to collagen production will be generated by the EcoeFISHent project. Of particular interest will be the aspect of being able to conduct analysis on pilot and semi-industrial plants, therefore with high efficiency and production capacity.

## Circular Economy Projects: The Case of EcoeFISHent

The FAO report “The State of World Fisheries and Aquaculture 2022” documents that the global consumption of aquatic foods has seen a substantial rise, now exceeding five times the amount consumed nearly 60 years ago. In 2019, the global intake of aquatic foods reached an estimated 158 million tons, up from 28 million tons in 1961. Factors such as increased supply, shifts in consumer preferences, technological advancements, and rising incomes have most significantly influenced per capita consumption. According to this, the amount of sidestreams is increasingly wasted instead of being valorized. In this light, global attention has increasingly focused on the issue of food by-products. Specifically, those generated by the fish supply chain have significant environmental and economic implications. This is because the environmental costs associated with food sidestreams extend beyond the disposal phase, encompassing processing and various earlier stages in its life cycle. Thus, reducing food sidestreams has the potential to greatly enhance environmental sustainability. This significance is particularly pronounced in the case of perishable goods like fresh seafood, which tend to generate higher waste volumes compared to other products.

EcoeFISHent, a project of the Horizon 2020 Program–Green Deal (G.A. ID 101036428), represents a good example on how to reduce and recycle fish sidestreams and enhance environmental sustainability. Indeed, the project is founded on the concept of a circular economy, representing an innovative and sustainable approach to resource management that seeks to maximize resource utilization and aims to demonstrate systematic solutions for “eco-efficient” territorial deployment.

The project involves 34 partners, from 7 countries (Bulgaria, France, Israel, Italy, Norway, Spain, and Kenya) with a particular focus geographical area in the Northwest of Italy corresponding to the Ligurian Sea and Central and Northern Tyrrhenian Sea, extending to Piedmont and Lombardy. However, the main goal of the project is to create a multi-level socio-economic and sustainable cluster that can be replicated in other European and extra-European regions.

In particular, within the fishing industry context, the “maximize resource utilization concept can be effectively applied to harness bioactive compounds from fish by-products, which are often overlooked.” In the light of achieving as much as possible value from these resources, one could consider the volume/profit pyramid value with the presence at the top of cosmetics and pharmaceutical products, since they require a small amount of bioactive molecules, and they have the highest value ref. Several compounds can be derived from marine sidestreams, including peptides, collagen, omega-3 fatty acids, and antioxidants. These compounds find applications in various technological sectors such as nutraceutical, cosmetics, bioengineering, and packaging. Collagen molecule, due to the biocompatibility and the physicochemical properties, represents one of the most versatile sidestreams-derived molecules. The project provides also the study of innovative solutions to bioactive extraction through the fishing sidestreams and co-products bioconversion by Black Soldier Fly to produce alternative soil fertilizer for agriculture, biodiesel oil for energy applications, and chitosan.

Furthermore, the EcoeFISHent project aims to increase awareness and possible solutions for the environment protection through a series of actions:

- The replacement of expanded polystyrene boxes, currently used as packaging, with expanded polylactic acid boxes, deriving from agro-sidestreams, and further recycled to obtain soil improver at the end of their life.
- The implementation of a “Stop Ghost Gear” program to remove the abandoned fishing nets from seabed marine protected area (e.g., Portofino Marine Protected Are—Liguria). The recovered fishing nets could be recycled and used as automotive components and bottles for cosmetic product packaging.
- The improvement of sustainable fishing techniques, which include selecting fishing tools, to preserve biodiversity and ecosystem equilibrium.

The flows of materials and data flows, generated within the cluster activities, will be supported and interconnected by applying big data analytic tools and through the advantages offered by the number of devices connected to the Internet of Things (IoT). This platform will be used to carry out a continuous assessment of sustainability, encompassing environmental, economic, and social aspects. Within the project, a Digital Twin Cluster will also be implemented. It will allow continuous monitoring of sustainability, while at the same time facilitating the replicability and transfer of the cluster model to other suitable geographical areas.

Lastly, the EcoeFISHent project addresses also societal challenges, supporting the local communities’ livelihoods relying on the blue economy.

## Conclusions

- As the main component of the extracellular matrix, collagen and its derivatives are widely used as nutritional, nutraceutical, and cosmetic compounds to promote tissue integrity and human health.
- The marine environment is a new and promising alternative source of collagen through the exploitation of fisheries discards.
- Numerous projects located in the Mediterranean basin are currently developing comprehensive strategies aimed at creating replicable and sustainable territorial clusters based on a multi-circular economy capable of unlocking the potential of marine by-products as collagen sources.
- This would ultimately lead to an increase in monetary income and well-being of the categories positioned at the beginning of the production chain.

## Data Availability

No datasets were generated or analysed during the current study.
